# Over 56.55% Faradaic efficiency of ambient ammonia synthesis enabled by positively shifting the reaction potential

**DOI:** 10.1038/s41467-018-08120-x

**Published:** 2019-01-21

**Authors:** Mengfan Wang, Sisi Liu, Tao Qian, Jie Liu, Jinqiu Zhou, Haoqing Ji, Jie Xiong, Jun Zhong, Chenglin Yan

**Affiliations:** 1 College of Energy, Key Laboratory of Advanced Carbon Materials and Wearable Energy Technologies of Jiangsu Province, Soochow University, 215006 Suzhou, China; 2 University of Electronic Science and Technology of China, 610054 Chengdu Sichuan, China; 3 Institute of Functional Nano and Soft Materials (FUNSOM), Jiangsu Key Laboratory for Carbon-Based Functional Materials and Devices, Soochow University, 215123 Suzhou, China

## Abstract

Ambient electrochemical N_2_ reduction is emerging as a highly promising alternative to the Haber–Bosch process but is typically hampered by a high reaction barrier and competing hydrogen evolution, leading to an extremely low Faradaic efficiency. Here, we demonstrate that under ambient conditions, a single-atom catalyst, iron on nitrogen-doped carbon, could positively shift the ammonia synthesis process to an onset potential of 0.193 V, enabling a dramatically enhanced Faradaic efficiency of 56.55%. The only doublet coupling representing ^15^NH_4_^+^ in an isotopic labeling experiment confirms reliable NH_3_ production data. Molecular dynamics simulations suggest efficient N_2_ access to the single-atom iron with only a small energy barrier, which benefits preferential N_2_ adsorption instead of H adsorption via a strong exothermic process, as further confirmed by first-principle calculations. The released energy helps promote the following process and the reaction bottleneck, which is widely considered to be the first hydrogenation step, is successfully overcome.

## Abstract (web summary)

While direct N_2_ reduction using electrochemistry offers an appealing method to obtain usable nitrogen, materials typically show poor activities and efficiencies. Here, authors demonstrate a single-atom catalyst, iron on N-doped carbon, to have dramatically enhanced N_2_ reduction efficiencies.

## Introduction

Known as the most common industrial chemical, ammonia (NH_3_) is provoking much attention as a highly efficient energy carrier with low liquefying pressure and high hydrogen density^1^. Its synthesis from the hydrogenation of atmospheric nitrogen (N_2_), however, is extremely difficult due to the inherent nature of the N–N triple bond^2^. Industrially, NH_3_ manufacture is still dominated by the long-standing Haber–Bosch process under harsh conditions, which consumes more than 1% of the energy supply on earth^3^. However, this energy-intensive process can only achieve a relatively low conversion ratio due to the unfavorable chemical equilibrium^4^. Therefore, to alleviate these conditions, it is of great significance to develop alternative routes for more efficient N_2_ fixation under milder conditions. In response, a worldwide gold rush has been triggered, and many pioneering methods have been reported, including biological catalysis^5,6^, photocatalysis^7,8^, and electrocatalysis^9,10^.

The electrochemical N_2_ reduction reaction (NRR) is emerging as a highly promising choice since it can be performed at atmospheric pressure and moderate temperature using a renewable electricity source^11,12^. From the thermodynamic view, the electrochemical NRR usually proceeds at similar potential to that required by the hydrogen evolution reaction (HER)^13^. In this case, the selectivity challenge comes from the competition between the binding of N_2_ (N_2_ + * → *N_2_) and H (H^+^ + *e*^−^ +* → *H), where * indicates the active site of the catalyst. Unfortunately, at relatively large applied potentials, most electrons and protons contribute to evolving hydrogen, accounting for the very low selectivity towards artificial synthesis of NH_3_ (ref. ^14^). To increase the NH_3_ selectivity, it would be desirable to fabricate an electrocatalytic system that could lower the free energy barrier of *N_2_ to compete with H for active sites and thus promote the NRR at low overpotentials. Once adsorbed N_2_ preoccupies the active sites instead of the undesirable coverage of hydrogen, the protons and electrons tend to attack the bound N_2_, leading to its hydrogenation and, finally, the synthesis of ammonia^13^. To date, although some catalysts have been synthesized to improve nitrogen adsorption and alleviate reaction barriers^15–17^, they still cannot efficiently separate the NRR from the HER and once the two reactions blur into each other, the Faradaic efficiency remains at very low levels. Accordingly, further efforts are needed to widen the potential gap between the NRR and the HER and to boost the NRR kinetics at low overpotentials to obtain reasonable performances.

Herein, we propose and demonstrate that a highly efficient and selective electrochemical NRR is indeed possible under ambient conditions by positively shifting the reaction potential. Starting with an onset potential of 0.193 V vs. reversible hydrogen electrode (RHE), the single-atom catalyst endowed the NRR with a superior Faradaic efficiency (56.55%) and a desirable yield rate (7.48 μg h^−1^ mg^−1^) at 0 V vs. RHE. An isotopic labeling experiment using ^15^N_2_ as the feeding gas was carried out and only a doublet coupling representing ^15^NH_4_^+^ was observed in the spectra, confirming that the produced NH_3_ entirely comes from the electroreduction of N_2_. Such excellent performance is a joint effect of a suppressed HER and an enhanced NRR. Molecular dynamics (MD) simulations suggest only a small energy barrier of 2.38 kJ mol^−1^ for the access of the N_2_ molecule to the single-atom Fe, benefiting efficient N_2_ delivery. The following adsorption is a strong exothermic reaction, as confirmed by density functional theory (DFT) calculations, and the energy released in this process could contribute to lowering the free energy barrier of the following hydrogenation, thus overcoming the reaction bottleneck of the whole process. On the other hand, considerably high energy barriers for *H adsorption as well as its conversion into H_2_ are observed in the catalytic system, indicating inferior HER activity.

## Results

### Synthesis and characterization of the Fe_SA_–N–C catalyst

**Fig. 1:**
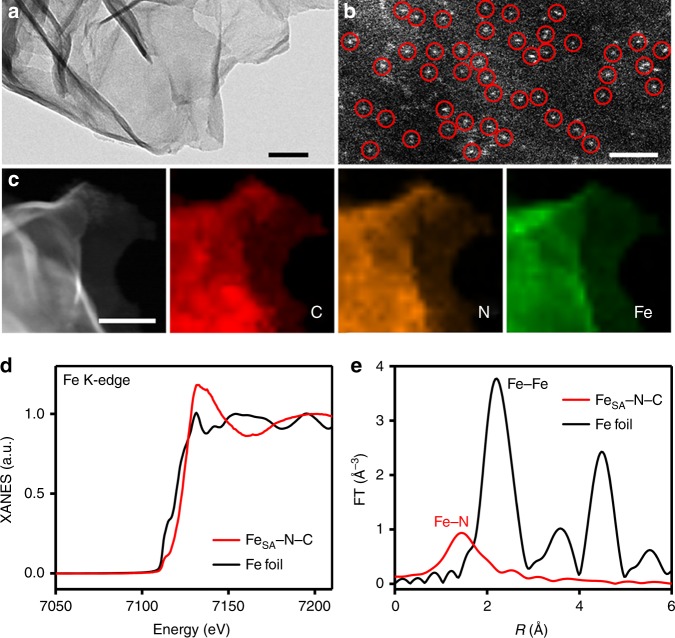
Physical characterization. **a** Transmission electron microscopy (TEM) image of single-atom dispersed Fe–N–C (Fe_SA_–N–C). Scale bar, 50 nm. **b** High-angle annular dark-field scanning transmission electron microscopy (HAADF-STEM) image of Fe_SA_–N–C. Single Fe atoms are highlighted by red circles. Scale bar, 2 nm. **c** High-resolution TEM (HRTEM) image and corresponding element maps showing the distribution of C (red), N (orange), and Fe (green). Scale bar, 50 nm. **d** X-ray absorption near-edge structure (XANES) spectra and **e** Fourier transform spectra at the Fe K-edge of Fe_SA_–N–C and Fe foil

The proof-of-concept single-atom dispersed Fe–N–C (Fe_SA_–N–C) catalyst was prepared by optimized modulation of the polypyrrole–iron coordination complex. Supplementary Fig. 1 and Fig. 1a indicate a graphene-like morphology of Fe_SA_–N–C, with no observable nanoparticle phase of iron. Aberration-corrected high-angle annular dark-field scanning transmission electron microscopy (HAADF-STEM) with subangstrom resolution was then conducted to elucidate the existing forms of Fe atoms. A large number of bright dots could be discerned in Fig. 1b, suggesting isolated iron atoms. The representative mapping images of the Fe_SA_–N–C sample in Fig. 1c further verify the uniform distribution of Fe atoms over the entire architecture along with N, which acted as anchor points for the Fe^18^. The Fe content is approximately 1.09 wt%, determined by inductively coupled plasma optical emission spectrometry analysis.

To further investigate the state of the Fe species at the atomic level, X-ray absorption near-edge structure (XANES) and extended X-ray absorption fine structure (EXAFS) analyses were carried out, with Fe foil as reference. In Fig. 1d, the absorption edge of Fe_SA_–N–C situates at more positive energy than does that of the Fe foil, implying that the isolated Fe single atoms bear positive charges. The Fourier transformed EXAFS curve of Fe_SA_–N–C only shows one distinct peak at approximately 1.44 Å, corresponding to the Fe–N scattering paths. Moreover, no obvious Fe–Fe peak (2.2 Å) or other peaks are observed (Fig. 1e). Iron atoms in Fe_SA_–N–C are thus confirmed to be stabilized by N atoms and atomically dispersed on the supports^19^. To probe the specific role of single Fe atoms, metal-free nitrogen-doped carbon (N–C) was synthesized for comparison (Supplementary Fig. 2). X-ray diffraction (XRD) patterns show no distinct differences between Fe_SA_–N–C and N–C, with two broad peaks assignable to the (002) and (101) planes of graphitic carbon (Supplementary Fig. 3a), which can also be verified by the *I*_D_/*I*_G_ value in Raman spectra (Supplementary Fig. 3b). Notably, no peaks related to metallic Fe or other Fe species are observed in the XRD pattern of Fe_SA_–N–C, further indicating the single-atomic nature of Fe.

### Electroreduction of N_2_ to NH_3_ on the Fe_SA_–N–C catalyst

The electrochemical measurements were conducted under ambient conditions using a gas-tight electrocatalytic system, wherein the Nafion membrane separated the two parts of the cell (Supplementary Fig. 4). To evaluate the electrocatalytic performance of the catalysts for the NRR, linear sweep voltammograms were first measured in an Ar- or N_2_-saturated solution. For this, 0.1 M KOH was chosen as the electrolyte. The HER in alkaline solution requires extra energy to break the water molecule, namely the strong covalent H–O–H bond, prior to hydrogen adsorption. Such an additional water dissociation step would make the entire process difficult to achieve at low overpotentials^20^. In an N_2_ environment, the current density of N–C increases obviously when the potential is more negative than −0.018 V vs. RHE, indicating the initiated NRR. For Fe_SA_–N–C, its onset potential shifted markedly to a more positive potential of 0.193 V vs. RHE, suggesting that Fe_SA_–N–C is much more active than is N–C for the NRR.

**Fig. 2:**
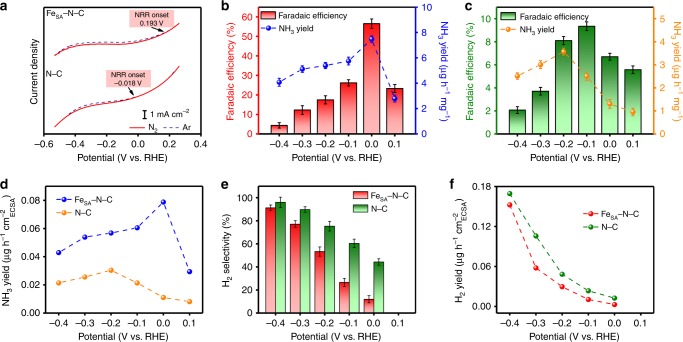
Electroreduction of N_2_ to NH_3_ at ambient conditions. **a** Linear sweep voltammograms of single-atom dispersed Fe–N–C (Fe_SA_–N–C) and nitrogen-doped carbon (N–C) in Ar-saturated (dashed line) or N_2_-saturated (solid line) 0.1 M KOH solution with a scan rate of 50 mV s^−1^. **b** NH_3_ Faradaic efficiencies and mass-normalized yield rates at each given potential of Fe_SA_–N–C. **c** NH_3_ Faradaic efficiencies and mass-normalized yield rates at each given potential of N–C. **d** Surface-area-normalized yield rate of NH_3_ production at different applied potentials on Fe_SA_–N–C and N–C. **e** H_2_ selectivity of Fe_SA_–N–C and N–C at different potentials. The error bars correspond to the standard deviations of measurements over three separately prepared samples under the same conditions. **f** Surface-area-normalized yield rate of H_2_ production at different applied potentials on Fe_SA_–N–C and N–C

The NRR activity of Fe_SA_–N–C was then systematically studied in 0.1 M KOH with continuous N_2_ bubbling at various potentials (Supplementary Fig. 5). The indophenol blue method was used to detect the produced ammonia. To avoid production loss by N_2_ blowing during the test, another glass tube filled with 0.001 M H_2_SO_4_ as the gas absorption liquid was set at the end of the cell. The total NH_3_ production yield was the summation of NH_3_ in the 0.1 M KOH and 0.001 M H_2_SO_4_ (Supplementary Figs. 6 and 7). Hydrazine was another possible product and was detected by the Watt and Chrisp method (Supplementary Figs. 8 and 9), but no hydrazine was produced in our work. The average ammonia yields and corresponding Faradaic efficiencies of Fe_SA_–N–C are plotted in Fig. 2b. Strikingly, the highest Faradaic efficiency of 56.55% with a yield rate of 7.48 μg h^−1^ mg^−1^ was achieved at 0 V vs. RHE. With negatively moving the applied potential, the adsorption of hydrogen species gradually outcompeted nitrogen on the electrode surface, leading to the HER as the primary reaction in the catalytic system. As a result, the Faradaic efficiency and the yield rate were cast into shade. To the best of our knowledge, such selectivity at 0 V vs. RHE is the highest among previously reported works under similar conditions (Supplementary Table 1).

To attest the superiority of Fe_SA_–N–C, the potentiostatic NRR tests for N–C were also conducted at the same potentials (Supplementary Fig. 10). As shown in Fig. 2c, for N–C, the highest NRR Faradaic efficiency is only 9.34%, which is a sixth of that of Fe_SA_–N–C. The double-layer capacitance (*C*_dl_) was calculated to determine the electrochemical active surface area (ECSA) of each sample (Supplementary Fig. 11). The surface-area-normalized yield rate of NH_3_ on Fe_SA_–N–C shows a greater advantage over the mass-normalized activity, corresponding to a 2.6-fold improvement compared with N–C towards the NRR (Fig. 2d). The hydrogen evolution selectivity was tested to support this huge performance gap (Fig. 2e). For Fe_SA_–N–C, with single-atom dispersed iron serving as active sites with a high selectivity of N_2_ adsorption with respect to H adsorption, inferior hydrogen evolution is observed at 0 vs. RHE. For N–C, its activity towards nitrogen reduction is relatively weak based on the narrow gap in the LSV curves of N_2_ and Ar-saturated environments, and most protons and electrons tend to evolve hydrogen. The surface-area-normalized activity of the HER was further calculated and compared in Fig. 2f. An H_2_ yield rate of 0.0029 μg h^−1^ cm_ECSA_^−2^ was obtained on Fe_SA_–N–C at 0 vs. RHE, which is less than a fourth of that on N–C (0.0125 μg h^−1^ cm_ECSA_^−2^), indicating that single Fe atoms could indeed retard the HER process. Based on the above results, we can conclude that the superior NRR activity of Fe_SA_–N–C derives from a joint effect of enhanced nitrogen reduction and suppressed hydrogen evolution.

**Fig. 3:**
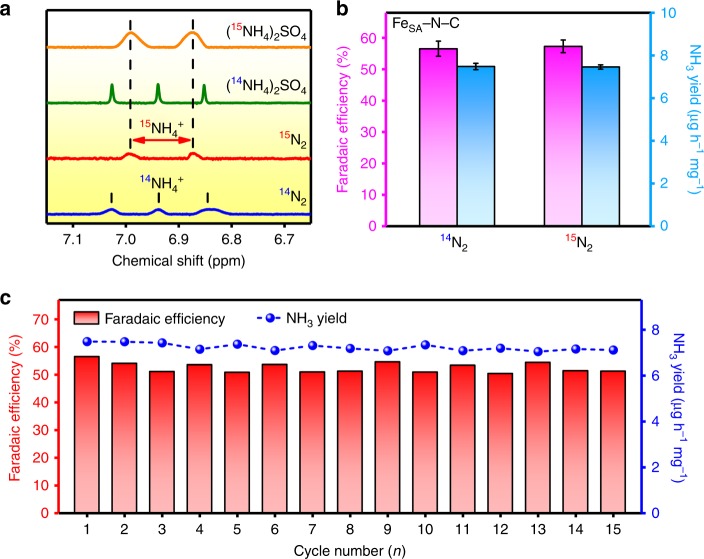
^15^N isotope labeling experiment and stability test. **a** ^15^N isotope labeling experiment. **b** Comparison of the Faradaic efficiency and ammonia yield rate using different feeding gases for the NRR at 0 V vs. reversible hydrogen electrode (RHE). The error bars correspond to the standard deviations of measurements over three separately prepared samples under the same conditions. **c** Faradaic efficiencies and NH_3_ yield rates of the single-atom dispersed Fe–N–C (Fe_SA_–N–C) calculated after consecutive recycling electrolysis in N_2_-saturated 0.1 M KOH at 0 V vs. RHE for 1 h

As a further proof that the produced NH_3_ originated from the feeding N_2_, ^15^N (99 atom% ^15^N) isotope labeling experiments were conducted^21^. Obviously, when ^15^N_2_ was supplied, no labeled ammonia was produced in the absence of an applied potential (Supplementary Fig. 12). After electrolysis at 0 vs. RHE, only doublet peaks representing ^15^NH_4_^+^ were observed in the ^1^H nuclear magnetic resonance (^1^H NMR) spectra of the electrolyte, while no triple coupling indicating ^14^NH_4_^+^ could be detected (Fig. 3a). Moreover, the measured Faradaic efficiency and yield rate match well with that determined using ^14^N_2_ as the feeding gas (Fig. 3b), confirming that the produced NH_3_ in our study entirely comes from the electroreduction of N_2_ catalyzed by Fe_SA_–N–C. In addition, control experiments without the Fe_SA_–N–C catalyst and with an Ar-saturated electrolyte were also performed as an alternative analytical method. As shown in Supplementary Fig. 13, the corresponding ultraviolet–visible (UV–vis) absorption spectra obtained using the indophenol blue method exhibit no apparent NH_3_ when using bare carbon paper as the working electrode. Similarly, upon conducting electrolysis on Fe_SA_–N–C-coated carbon paper in an Ar-saturated solution, no NH_3_ was detected, further indicating a reliable NH_3_ production data_._ The stability of the catalyst is important for practical use, and it was investigated by consecutive recycling electrolysis at 0 V vs. RHE. For Fe_SA_–N–C, no obvious change could be observed in the total current density during 15 consecutive cycles (Supplementary Fig. 14) nor in the ammonia Faradaic efficiency and yield rate (Fig. 3c), suggesting its excellent stability for ambient ammonia synthesis. Careful examination of SEM, TEM, and HAADF-STEM images of Fe_SA_–N–C show that the atomically dispersed Fe atoms were still anchored the N-doped carbon nanosheet without any agglomeration (Supplementary Figs. 15 and 16). The catalyst phase property also shows no obvious change, as confirmed by further characterization with XRD (Supplementary Fig. 17), indicating the robustness of Fe_SA_–N–C towards ammonia synthesis.

## Discussion

Emerging cutting-edge operando techniques are considered critical for giving insight into the intrinsic mechanism of catalytic processes compared with ex-situ measurements. In-situ XANES with ultrasensitivity to the electronic properties of the metal was thus conducted under operation conditions to probe if single-atom Fe was the actual active phase for the NRR (Supplementary Fig. 18). Potentiostatic testing was carried out at 0 V vs. RHE and the XANES results of the Fe K-edge were collected at different times, as shown in Supplementary Fig. 19. Obviously, once the reaction proceeds, the white-line peak shifted from 7133 eV to a lower energy of 7131 eV after 15 min, suggesting a decreased valence state of Fe. During the NRR process, N_2_ molecules are fixed to bond with Fe sites by donating electrons to the unoccupied *d* orbitals of Fe^22,23^, thus accounting for the negative shift of its energy position. As the reaction goes on, the adsorption of nitrogen and the desorption of ammonia constantly occur and reach a dynamic balance; thus, the adsorption edge of Fe shows no distinct change from 15 to 30 min. The XANES and EXAFS profiles obtained after the NRR remain almost the same as those obtained before the reaction (Supplementary Fig. 20), which is consistent with the lack of change in the HAADF-STEM images and XRD patterns of the fresh and spent electrocatalysts, again confirming the stability of the single-atom Fe.

**Fig. 4:**
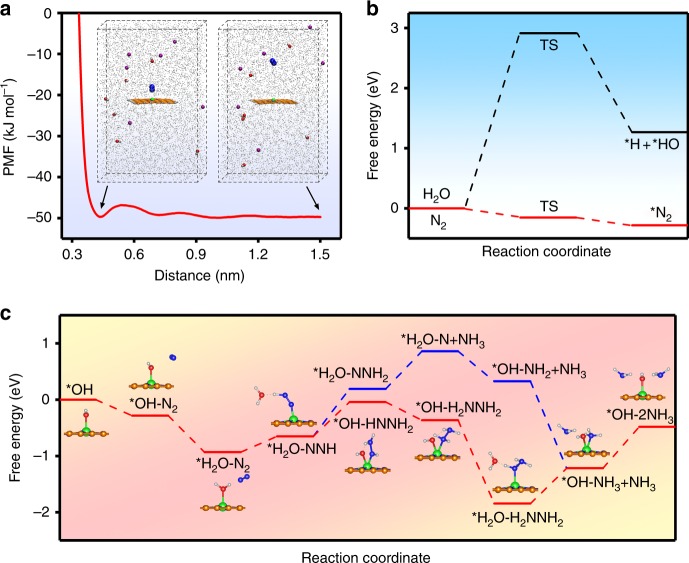
Computational studies. **a** Potential of the mean force (PMF) for N_2_ adsorption on the single-atom dispersed Fe–N–C (Fe_SA_–N–C) in 0.1 M KOH, inset: Molecular dynamics (MD) simulation snapshots at 0.45 and 1.5 nm, with N_2_, blue spheres; H_2_O, gray dots; OH^−^, red and white sticks; K^+^, purple spheres. **b** Calculated energy barriers of the adsorption of hydrogen and nitrogen. **c** Free energy diagram for the nitrogen reduction reaction (NRR) on the Fe_SA_–N–C catalyst at U = 0 V. Inset models represent the corresponding adsorbates. The orange, blue, red, green, and gray spheres represent C, N, O, Fe, and H atoms, respectively

Computational studies were performed to understand the unique activity of the NRR on the Fe_SA_–N–C catalyst in alkaline medium (Supplementary Fig. 21). The adsorption behavior of N_2_ towards single-atom Fe was first explored by MD simulations. Umbrella sampling was performed to quantify the free energy barrier of N_2_ access (Supplementary Fig. 22), and the potential of the mean force (PMF) as a function of the distance is shown in Fig. 4a. Clearly, starting from a distance of 1.5 nm, there is only a small energy barrier of 2.38 kJ mol^−1^ at approximately 0.54 nm for the approaching of N_2_ molecule. As a result, the N_2_ molecules tend to accumulate at approximately 0.45 nm away from the Fe site, leading to a localized high concentration, which would facilitate the following adsorption and thus benefit the whole NRR process. The competing adsorption between *N_2_ and *H was then evaluated by DFT calculations in Fig. 4b. In our case, *H comes from water dissociation and the energy barrier of this step is as high as 2.91 eV, representing negligible *H adsorption. Even if *H is adsorbed, its desorption to form H_2_ is still an endothermic reaction, with a much stronger Gibbs free energy change than those of common transition and noble metals^24^, as shown in Supplementary Fig. 23. Therefore, the HER kinetics are particularly sluggish on the Fe_SA_–N–C surface. In contrast, it was found that nitrogen adsorption undergoes a downhill process and the binding Gibbs free energy of *N_2_ is as low as −0.28 eV, suggesting a significantly exothermic process. The usual weakness of the low absorbability of nitrogen is thus overcome^25^.

Notably, considering the experimental environment of N_2_-saturated 0.1 M KOH, the solvent effect on NRR cannot be ignored and the adsorption energies of *H_2_O, *N_2_, *OH^−^, and *K^+^ (Supplementary Fig. 24) are compared in Supplementary Table 3. It turns out that *OH^−^ is the easiest to adsorb onto Fe_SA_–N–C; thus, the Gibbs free energy for each step of the NRR was calculated based on the Fe-*OH^−^ structure. The computed energy profiles are depicted in Fig. 4c, and the alternating pathway (Supplementary Fig. 25) is more feasible than the distal pathway (Supplementary Fig. 26), as hydrogenation occurs preferentially between the two N atoms. The hydrogen tends to first attack the adsorbed *OH^−^ and the reaction process from *OH^−^ to *H_2_O-N_2_ is completely barrier-less (Fig. 4c). This thermodynamically favorable process could contribute to the activation of the following hydrogenation steps. According to the so-called hot atom mechanism, the molecule’s vibrational energy level can be enhanced by the energy released in the nitrogen adsorption process^26^. As a result, the reaction barrier of the following nitrogen activation is reduced. The reaction Gibbs free energy of the first hydrogenation step (*H_2_O-N_2_ + H^+^ + *e*^−^ → *H_2_O-NNH), which is usually considered the rate-determining step of the whole process, is calculated to be only 0.28 eV uphill after nitrogen adsorption, which is much lower than that of the previously reported noble metal catalyst^11^. The reaction bottleneck is shifted to the last step (*OH-NH_3_ + NH_3_ + H^+^ + *e*^−^ → *OH + 2NH_3_) and the overall reaction barrier is dramatically reduced. The DFT-calculated free energetics of the HER and NRR rationalize the electrochemical measurements and we can conclude that the desirable performance derives from the preferential nitrogen adsorption and its exothermic effect on the following nitrogen activation.

In summary, we have shown that positively shifting the reaction potential of the NRR enabled a single-atom dispersed Fe–N–C catalyst to achieve a Faradaic efficiency of 56.55% and a desirable yield rate of 7.48 μg h^−1^ mg^−1^ under ambient conditions. Comparative experiments indicate both enhanced NRR and suppressed HER performance. The promoted NRR process starts with a very positive onset potential of 0.193 V vs. RHE and achieves the best performance at 0 V vs. RHE. To determine reliability of the NH_3_ production data, an ^15^N isotopic labeling experiment using ^15^N_2_ as the feeding gas was carried out, and all the produced NH_3_ were confirmed to come from the electroreduction of N_2_ catalyzed by Fe_SA_–N–C. In addition, during consecutive recycling, no obvious change can be observed in the ammonia Faradaic efficiency and yield rate, indicating the excellent stability of the catalyst. The specific mechanism of this excellent activity is unveiled by the MD and DFT calculations. The Fe_SA_–N–C structure could effectively attract the access of nitrogen molecules, with a small energy barrier of 2.38 kJ mol^−1^. The obtained localized high concentration of N_2_ would favor the following adsorption with a particularly low binding Gibbs free energy of −0.28 eV. Moreover, such an exothermic process could provide energy for the following hydrogenation, as suggested by the hot atom hypothesis. As a result, the overall reaction barrier is dramatically reduced, and a highly selective ammonia synthesis is realized.

## Methods

### Reagents

Pyrrole (C_4_H_5_N, 98%) was purchased from Sigma-Aldrich. Ferrous chloride tetrahydrate (FeCl_2_·4H_2_O, ≥99.0%), hydrogen peroxide solution (H_2_O_2_, 30 wt% in H_2_O), sodium chloride (NaCl, 99.5%), ammonium persulfate ((NH_4_)_2_S_2_O_8_, ≥ 98%), potassium hydroxide (KOH, 95%), phenol (C_6_H_6_O, ≥99.5%), sodium nitroferricyanide dihydrate (C_5_FeN_6_Na_2_O·2H_2_O, 99.0%), sodium citrate dehydrate (C_6_H_5_Na_3_O_7_·2H_2_O, 99.0%), sodium hydroxide (NaOH, 96%), salicylic acid (C_7_H_6_O_3_, 99.5%), sodium hypochlorite solution (NaClO, available chlorine≥5.0%), ammonium chloride (NH_4_Cl, 99.5%), *p*-dimethylaminobenzaldehyde (C_9_H_11_NO, 99%) were purchased from Aladdin. Hydrochloric acid (HCl, 36.0–38.0%), sulfuric acid (H_2_SO_4_, 95.0–98.0%), ethyl alcohol (C_2_H_5_OH, 75%), hydrazine monohydrate (N_2_H_4_·H_2_O, ≥85.0%) were purchased from Sinopharm Chemical Reagent Co., Ltd. All chemical reagents were used without further purification.

### Catalyst preparation

To prepare Fe_SA_–N–C, 2 ml pyrrole was adequately dispersed in deionized water by ultrasonic vibration. Excess ferrous chloride and hydrogen peroxide were added, and the reaction lasted several hours until a bright yellow and transparent solution was obtained. Sodium chloride was further dissolved in the solution to be used as a template. The product was collected by freeze-drying to obtain brown powders. The material was then placed in a ceramic boat and carbonized at 600 °C for 2 h under argon protection. After removing the salt templates through sufficient washing with a lot of water and ethanol, the products were dried in a vacuum at 60 °C overnight prior to use. To prepare N–C, 2 ml pyrrole was adequately dispersed in deionized water by ultrasonic vibration. Sodium chloride was dissolved in the solution, followed by the addition of 1 ml 1 M HCl. Then, (NH_4_)_2_S_2_O_8_ solution was dropwise added into the system under vigorous stirring to start the pyrrole polymerization. The solution was freeze-dried after the reaction and was kept in an ice/water bath for 24 h. The obtained material was placed in a ceramic boat and carbonized at 600 °C for 2 h under argon protection. After removing the salt templates through sufficient washing with a lot of water and ethanol, the samples were dried in a vacuum at 60 °C overnight to obtain black powders.

### Physical characterization

The morphology was studied via a field emission scanning electron microscope (FESEM, SU8010, Japan) and a field emission transmission electron microscope (FETEM, FEI Tecnai G2 F20 S-TWIN TMP, Hongkong). The dispersion of single Fe atoms was characterized by atomic-resolution high-angle annular dark-field scanning transmission electron microscopy on a JEOL JEM-ARM200F instrument equipped with a probe spherical aberration corrector. The iron concentrations of the samples were determined via inductively coupled plasma atomic emission spectroscopy. The EXAFS spectra (Fe K-edge) were collected at 1W1B station in the Beijing Synchrotron Radiation Facility (BSRF). The storage rings of BSRF were operated at 2.5 GeV with a maximum current of 250 mA. Using an Si (111) double-crystal monochromator, data collection was carried out in transmission mode using the ionization chamber for the Fe foil and in fluorescence excitation mode using a Lytle detector for Fe_SA_–N–C. All spectra were collected in ambient conditions. The composition of the catalysts was characterized by XRD patterns (D8 Advance, Bruker) and Raman spectroscopy (HR evolution, Horiba Jobin Yvon, France).

### Cathode preparation

Typically, 1 mg of catalyst and 5 μl of Nafion solution (Alfa Aesar, 5 wt%) were dispersed in 100 μl of ethanol by sonicating for at least 1 h to form a homogeneous ink. Then, the catalyst ink was loaded onto carbon paper with an area of 1 × 1 cm^2^ and dried in N_2_ atmosphere at 80 °C for 1 h. The obtained mass loading was 1 mg cm^−2^.

### Electrochemical NRR measurements

The reduction of N_2_ gas was performed in a two-compartment cell at room temperature, which was separated by a Nafion 211 membrane (DuPont). Before the NRR tests, the Nafion membrane was pretreated by successive heating in H_2_O_2_ (5%) aqueous solution at 80 °C for 1 h and in ultrapure water at 80 °C for another 1 h. A conventional three-electrode system in an electrochemical workstation was used to conduct electrochemical measurements, with Fe_SA_–N–C or N–C as the working electrode, Pt foil as the counter electrode, and Ag/AgCl (4 M KCl) as the reference electrode. All potentials were converted to the RHE scale via calibration. Pure N_2_ was continuously fed into the cathodic compartment, with a properly positioned sparger to ensure that the entire cathode was hit by the N_2_ gas bubbles during the experiments. The electrochemical NRR was tested in N_2_-saturated 0.1 M KOH (30 ml in each cell compartment) at ambient temperature and pressure. The linear sweep voltammetry was scanned at a rate of 50 mV s^−1^. The potentiostatic tests were tested at different potentials including 0.1, 0, −0.1, −0.2, −0.3, and −0.4 V vs. RHE. To avoid the loss of produced NH_3_ by N_2_ blowing during the test, another glass tube filled with 0.001 M H_2_SO_4_ (30 ml) as the gas absorption liquid was set at the end of the cell. The total NH_3_ production yield was the summation of the NH_3_ in the 0.1 M KOH and 0.001 M H_2_SO_4_. After the electrochemical reduction reaction, the electrolyte and gas absorption liquid were both collected and analyzed by chromogenic reactions for qualitative measurements. For comparison, potentiostatic tests in Ar-saturated 0.1 M KOH were also conducted in this work.

### Determination of the produced ammonia

The concentration of the produced ammonia in the 0.1 M KOH and 0.001 M H_2_SO_4_ was determined by the indophenol blue method^7^. For the 0.1 M KOH case, 10 ml of the electrolyte after the NRR potentiostatic test was taken out, and 0.4 ml of the solution containing 5 g of phenol in 50 ml of ethanol, and 0.4 ml of 0.5 wt% C_5_FeN_6_Na_2_O (sodium nitroferricyanide) was successively added. Then, 1 ml of oxidizing solution containing 10 g of sodium citrate dehydrate, 0.5 g of sodium hydroxide and 10 ml of sodium hypochlorite in 50 ml of Millipore water was also added into the above solution. For the 0.001 M H_2_SO_4_ case, 2 ml of the electrolyte after the NRR potentiostatic test was taken out. Next, 2 ml of a 1 M NaOH solution containing salicylic acid and sodium citrate dehydrate was added. Then, 1 ml of 0.05 M NaClO and 0.2 ml of 1 wt% C_5_FeN_6_Na_2_O (sodium nitroferricyanide) were also added into the above solution. The UV–Vis absorption spectrum was measured after the mixture was stored in darkness at room temperature for 3 h. The concentration of indophenol blue was measured according to the absorbance at a wavelength of 655 nm. Standard NH_4_Cl solutions at a series of concentrations in 0.1 M KOH and 0.001 M H_2_SO_4_ were prepared to build the calibration curves and quantify the produced ammonia.

### Determination of the produced hydrazine

The concentration of the produced hydrazine in the 0.1 M KOH and 0.001 M H_2_SO_4_ was determined by the Watt and Chrisp method^27^. In both cases, a mixture of ethanol (300 ml), HCl (concentrated, 30 ml), and *p*-dimethylaminobenzaldehyde (5.99 g) was used as a color reagent. For this, 5 ml of the residual electrolyte after the NRR potentiostatic test was taken out, and 5 ml of the above-prepared color reagent was successively added. The resulting solution was stirred for 10 min, and its absorbance was measured at a wavelength of 455 nm. Standard hydrazine monohydrate solutions at a series of concentrations in 0.1 M KOH and 0.001 M H_2_SO_4_ were prepared to build the calibration curves and quantify the produced hydrazine.

### Determination of the HER productions

The H_2_ was manually sampled and analyzed by gas chromatography (GC, Agilent 7890B).

### Calculation of the equilibrium potential

The standard potential for the NRR in alkaline medium was calculated from the standard Gibbs energy of formation at 298.15 K.

1 $$\begin{array}{c}{\mathrm {N}_2}\left( {\mathrm {g}} \right) + {\mathrm{8H}}_2{\mathrm {O}} + 6{\mathrm{e}}^ - \to {\mathrm {2NH}_4{\mathrm {OH}}}\left( {{\mathrm {aq}}} \right) + {\mathrm {6OH}}^ - \quad \\ \Delta G^{\mathrm{o}} = 426.38\,{\mathrm {kj}\,{\mathrm {mol}^{ - 1}}}\end{array},$$

2 $$\begin{array}{c}E^\circ = - \Delta G^{\mathrm{o}}/nF\\ = - 0.737\,{\mathrm{V}}\,{\mathrm{versus}}\,{\mathrm{Standard}}\,{\mathrm{Hydrogen}}\,{\mathrm{Electrode}}\,\left( {{\mathrm{vs}}{\mathrm{.}}\,{\mathrm{SHE}}} \right)\end{array},$$

where *n* is the number of transferred electrons (6) and *F* is the Faraday constant (96,485 C mol^−1^).

The thermodynamic equilibrium potential under the reaction conditions was calculated according to the Nernst equation, assuming 1 atm of N_2_ in the solution.

3 $$\begin{array}{c}E = E^\circ - RT/6F \times {\mathrm {ln}}\left[ {c^2({\mathrm {NH}_4{\mathrm {OH}}}) \times c^6\left( {{\mathrm {OH}}^ - } \right)} \right]\\ \hskip -32pt + 0.059 \times {\mathrm {pH}}\left( {{\mathrm {vs.}\, {\mathrm {RHE}}}} \right)\end{array},$$

where *R* is the gas constant (8.314 J mol^−1^ K^−1^), *T* is the temperature in Kelvin (298.15 K), *F* is the Faraday constant (96,485 C mol^−1^), *c*(OH^−^) is the hydroxide concentration (0.1 M), and the pH is 13. Assuming a *c*(NH_4_OH) of 10^−7^ M in the solution, the corresponding thermodynamic equilibrium potentials is determined to be 0.227 V vs. RHE.

### Faradaic efficiency and the yield rate

The Faradaic efficiency (FE) and mass-normalized yield rate of NH_3_ were calculated as below:

4 $${\mathrm {FE}}\left( {{\mathrm {NH}_{3}}} \right) = [3F \times c\left( {{\mathrm {NH}_{3}}} \right) \times V]/Q,$$

5 $${\mathrm {Yield}}\,{\mathrm {rate}}_{{\mathrm {mass}}}\left( {{\mathrm {NH}_{3}}} \right) = \left[ {17c\left( {{\mathrm {NH}_{3}}} \right) \times V} \right]/\left( {t \times m} \right),$$

where *F* is the Faraday constant (96,485 C mol^−1^), *t* is the electrolysis time (1 h), *m* is the loading mass of the catalysts (1 mg), *Q* is the total charge passed through the electrode, *V* is the volume of the electrolyte, and *c*(NH_3_) is the measured ammonia concentration.

The electrochemical double-layer capacitance (*C*_dl_) of the materials was measured to determine their electrochemical surface area (ECSA) using the cyclic voltammograms (CVs) in a small potential range with no faradic processes between 0.9 and 1.0 V vs. RHE. The plotted current density against scan rate has a liner relationship and its slope is twice the *C*_dl_. The ECSA can then be calculated as below:

6 $$A_{{\mathrm {ECSA}}} = C_{{\mathrm {dl}}}\,{\mathrm {of}}\,{\mathrm {catalyst}}\,\left( {{\mathrm {mF}\,{\mathrm {cm}^{ - 2}}}} \right)/40\,\mu {\mathrm F}\,{\mathrm {cm}^{ - 2}\,{\mathrm {per}\,{\mathrm {cm}_{\mathrm {{ECSA}}}}}}^2.$$

The surface-area-normalized activity of NH_3_ was calculated as below:

7 $${{\mathrm{Yield}}\,{{\mathrm{rate}}_{\mathrm{{ECSA}}}}}\left( {{\mathrm{NH}}_{3}} \right) = \left[ {17c\left( {{{{\rm{NH}}}_{3}}} \right) \times V} \right]/\left( {t \times A_{{\mathrm {ECSA}}}} \right).$$

The Faradaic efficiency of H_2_ was calculated as below:

8 $${\mathrm {FE}}\left( {{\mathrm {H}_{2}}} \right) = 2Fv\left( {{\mathrm {H}_{2}}} \right)Gp_0/RT_0i_{{\mathrm {total}}},$$

where *v*(H_2_) is the volume concentration of H_2_ in the exhaust gas from the electrochemical cell (GC data), *G* is the gas flow rate (ml min^−1^ at room temperature and ambient pressure), *i*_total_ is the steady-state cell current, *p*_0_ = 1.01 × 10^5^ Pa, and *R* = 8.314 J mol^−1^ K^−1^.

The surface-area-normalized activity of H_2_ was calculated as below:

9 $${\mathrm {Yield}\,{\mathrm {rate}_{\mathrm {{ECSA}}}}}\left( {{\mathrm {H}_{2}}} \right) = \left[ {Q \times {\mathrm {FE}}\left( {{\mathrm {H}_{2}}} \right) \times M_{\mathrm{ r}}\left( {{\mathrm {H}_{2}}} \right)} \right]/\left( {e \times N_{\mathrm {A}} \times 2} \right) \times \left( {t \times A_{{\mathrm {ECSA}}}} \right),$$

where *M*_r_(H_2_) is the relative molecular mass of H_2_, *e* is the electron charge (1.602 × 10^−19^ C), and *N*_A_ is the Avogadro constant (6.02 × 10^23^ mol^−1^).

### ^15^N isotopic labeling experiment

^15^N_2_ (Wuhan Newradar Special Gas Co. Ltd 99 atom% ^15^N) was used as the feeding gas in the labeling experiment. A low-velocity gas flow system was adopted due to the limited supply and expense of ^15^N_2_. After electrolysis at 0 V vs. RHE for 10 h, 10 ml of the electrolyte was taken out, and its pH was adjusted to 3 by adding 0.5 M H_2_SO_4_. Then, the solution was concentrated to 2 ml and 0.9 ml of the resulting liquid was taken out, followed by adding 0.1 ml of D_2_O as an internal standard. The produced ammonia was quantified using ^1^H nuclear magnetic resonance measurements (^1^H NMR; Agilent 600 MHz).

### In-situ XAS characterization for NRR

In-situ and ex-situ XAS data were collected at room temperature at the BL14W1 beamline of Shanghai Synchrotron Radiation Facility (SSRF). Energy calibration was carried out with a Fe foil standard using transmission mode, while the Si (111) monochromator scanned the incident X-ray photon energy through the Fe K absorption edge. All spectra of the samples were measured in fluorescence mode. The special three-electrode system was assembled using a tailor-made mold with a window for X-ray penetration. The working electrode was prepared by casting Fe_SA_–N–C onto carbon paper. A graphite rod was used as the counter electrode, and Ag/AgCl (4 M KCl) was used as the reference electrode. The electrolyte was N_2_-saturated 0.1 M KOH. Potentiostatic testing at 0 V vs. RHE was used for in-situ XAS characterization using the CHI660E (Shanghai Chenhua instrument Co., Ltd) electrochemical workstation.

### Computational method and model

The umbrella-sampling method was used to calculate the PMF for the adsorption of nitrogen molecules onto Fe_SA_–N–C. The Fe_SA_–N–C was placed at the center of a simulation box (4 nm × 4 nm × 6 nm) and perpendicular to the *z*-axis, with one N_2_ molecule right above the center Fe atom at a distance of 1.5 nm. Then, the box was solvated with KOH solution (containing 6 K^+^, 6 OH^–^ and 3000 water molecules). The entire system was energy-minimized (EM) and *NPT*-equilibrated (constant number of atoms, pressure, and temperature) at 298 K and 1 bar for 1 ns.

Subsequently, the N_2_ molecule was pulled towards the Fe atom along the *z*-axis at a rate of 0.01 nm ps^–1^ with a force constant of 10^5^ kJ mol^–1^ nm^–2^. The trajectory was extracted, and a series of configurations was picked out, with the center of mass position of the N_2_ molecule in adjacent configurations at 0.03 nm. These consecutive configurations were used as windows for umbrella sampling for 8 ns at *NPT* (298 K, 1 bar). The temperature and pressure were controlled by the Nose-Hoover thermostat and Parrinello-Rahman barostat, respectively.

A harmonic force constant of 10^4^ kJ mol^–1^ nm^–2^ in all directions was employed to restrain the Fe_SA_–N–C. For the N_2_ molecule, a harmonic force constant of 10^6^ kJ mol^–1^ nm^–2^ was employed during EM and NPT equilibration in all directions but only in the *x*–*y* directions during pulling and umbrella sampling. During umbrella sampling, a harmonic force constant of 10^5^ kJ mol^–1^ nm^–2^ was employed in the *z*-direction. The weighted histogram analysis method was used to obtain the PMF curve.

The DFT calculations were conducted based on the Cambridge Sequential Total Energy Package (CASTEP). The exchange-correlation function under the generalized gradient approximation (GGA) with norm-conserving pseudopotentials and the Perdew–Burke–Ernzerhof function was adopted to describe the electron–electron interaction. An energy cutoff of 750 eV was used, and a k-point sampling set of 5 × 5 × 1 was tested to be converged. A force tolerance of 0.01 eV Å^−1^, an energy tolerance of 5.0 × 10^−7^ eV per atom, and a maximum displacement of 5.0 × 10^−4 ^Å were considered. Each atom in the storage models is allowed to relax to the minimum enthalpy without any constraints. The vacuum space along the *z*-direction is set to be 15 Å. The intermediates have been absorbed onto the Fe atom of the substrate.

The complete linear and quadratic synchronous transit method search protocol, the self-consistent field tolerance of 5.0 × 10^−7^ eV, and root mean square convergence of 0.02 eV/Å were set for transition states.

The adsorption energy Δ*E* of the A group on the surface of the substrates was defined as

10 $$\Delta E = E_{ \ast {\mathrm A}}-\left( {E_ \ast + E_{\mathrm A}} \right),$$

where *A and * denote the adsorption of the A group onto the substrates and the bare substrates and, *E*_A_ denotes the energy of the A group.

The Gibbs free energy change (Δ*G*) of each chemical reaction is calculated by

11 $$\Delta G = \Delta E + \Delta {\mathrm {ZPE}}-T\Delta S + \Delta G_U + \Delta G_{{\mathrm {pH}}},$$

where *E*, ZPE, *T*, and *S* denote the calculated total energy, zero-point energy, temperature, and entropy, respectively. Δ*G*_*U *_= –*eU* (*U* is the potential measured against a normal hydrogen electrode) and Δ*G*_pH _= –*kBT*ln(10) × pH. Here, *T* = 300 K and pH = 13 are considered.

## Supplementary information

Supplementary Information

## Data Availability

The data that support the findings of this study are available from the corresponding author upon reasonable request.
