# Supplementary material for: Over 56.55% Faradaic efficiency of ambient ammonia synthesis enabled by positively shifting the reaction potential
Source: Nat Commun. 2019 Jan 21;10:341. doi: 10.1038/s41467-018-08120-x (PMC6341113; doi:10.1038/s41467-018-08120-x)
Supplement: Supplementary file 1 — Supplementary Information [file 41467_2018_8120_MOESM1_ESM.pdf]

Supplementary Information

Over 56.55% Faradaic efficiency of ambient ammonia synthesis enabled by positively shifting the reaction potential

Wang et al.

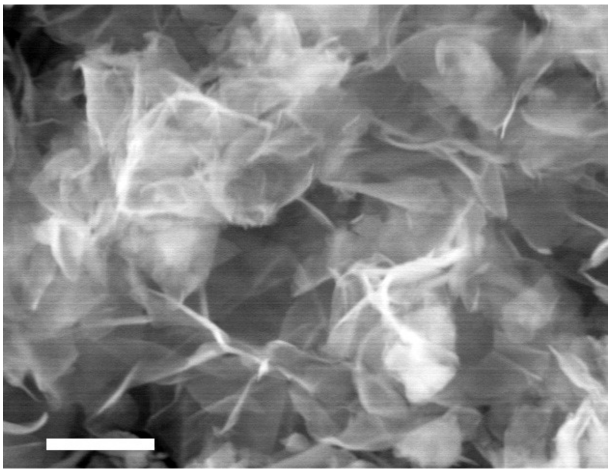

Supplementary Figure 1. SEM image of Fe_{SA}-N-C. Scale bar, 200 nm.

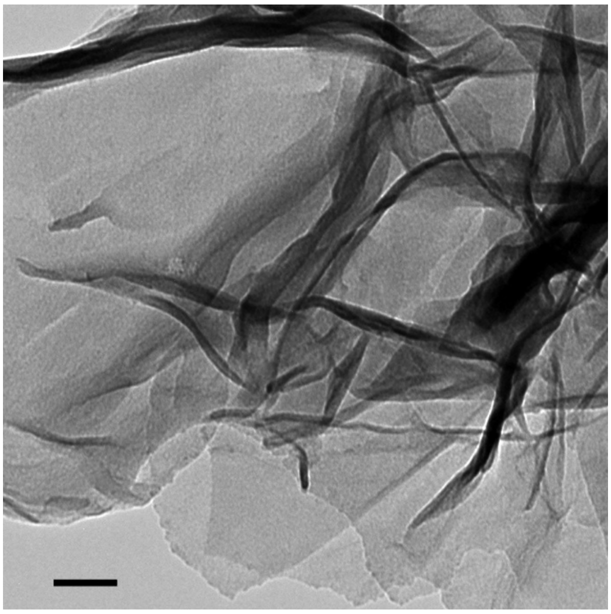

Supplementary Figure 2. TEM image of N-C. N-C also exhibits a graphene-like morphology and shows no difference compared with Fe_{SA}-N-C. Scale bar, 50 nm.

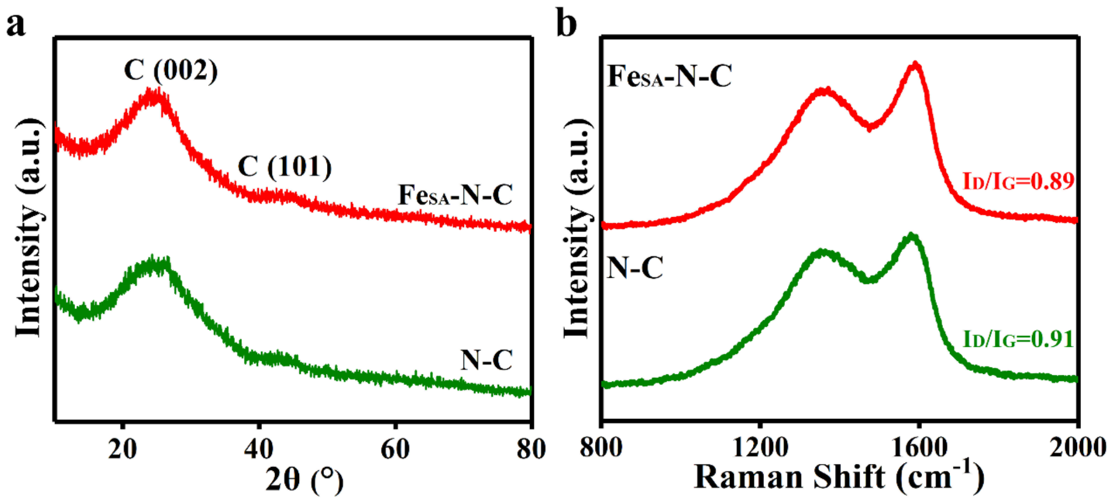

Supplementary Figure 3. Structural characterizations of the Fe_{SA}-N-C and N-C. **a** XRD patterns and **b** Raman spectra of Fe_{SA}-N-C and N-C. XRD results show no distinct differences between Fe_{SA}-N-C and N-C, with two broad peaks assignable to the (002) and (101) planes of graphitic carbon. Notably, no peaks related to metallic Fe or other Fe species are observed in XRD pattern of Fe_{SA}-N-C, further indicating the single-atomic nature of Fe. The graphitic carbon plane can also be verified by Raman spectra, with I_D/I_G values of 0.89 and 0.91 for Fe_{SA}-N-C and N-C, respectively.

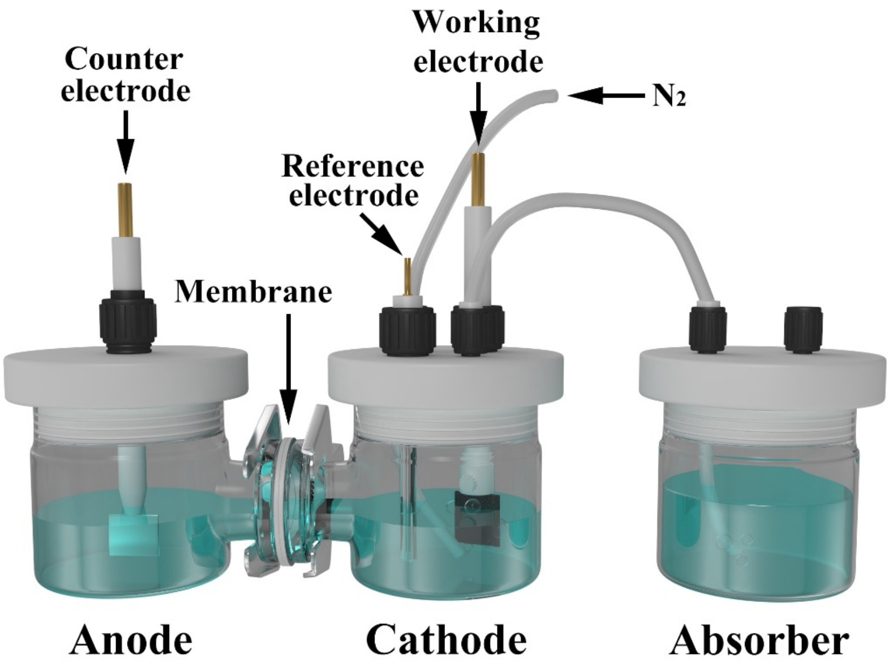

Supplementary Figure 4. Schematic for electrocatalytic NRR.

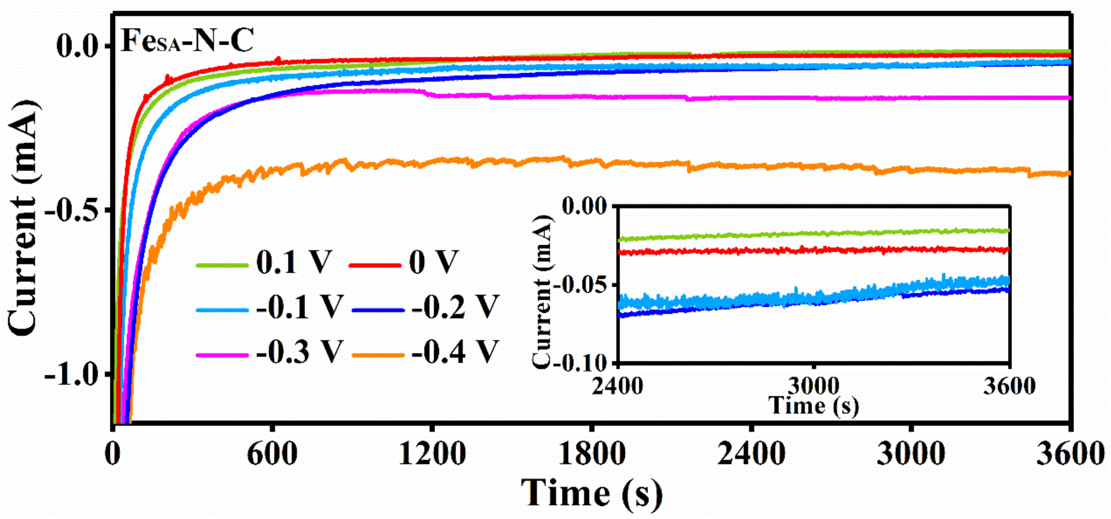

Supplementary Figure 5. Chronoamperometry results of Fe$_{SA}$-N-C at the corresponding potentials, inset: enlarged view of the curves from -0.2 to 0.1 V vs. RHE.

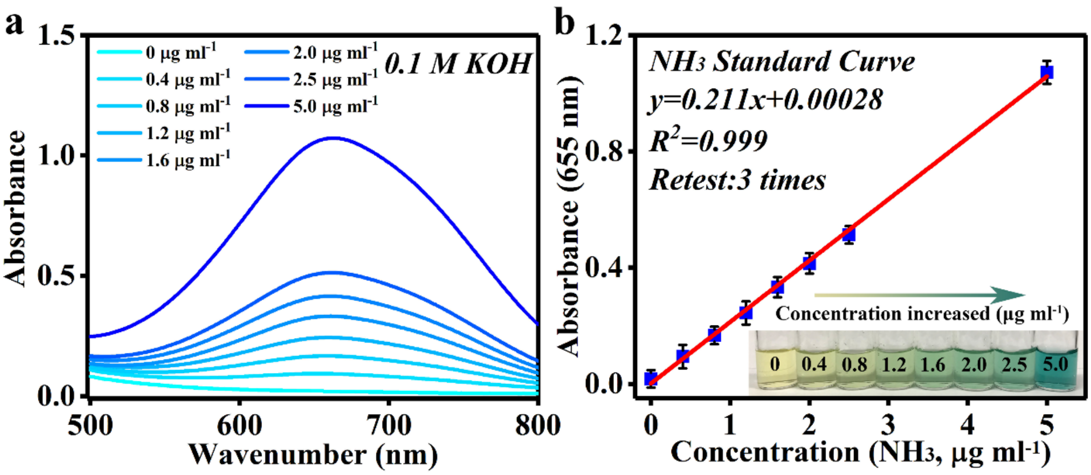

Supplementary Figure 6. Determination of the produced ammonia in 0.1 M KOH. **a** The UV-Vis absorption spectra and **b** corresponding calibration curves for the colorimetric NH_3 assay using the indophenol blue method in 0.1 M KOH.

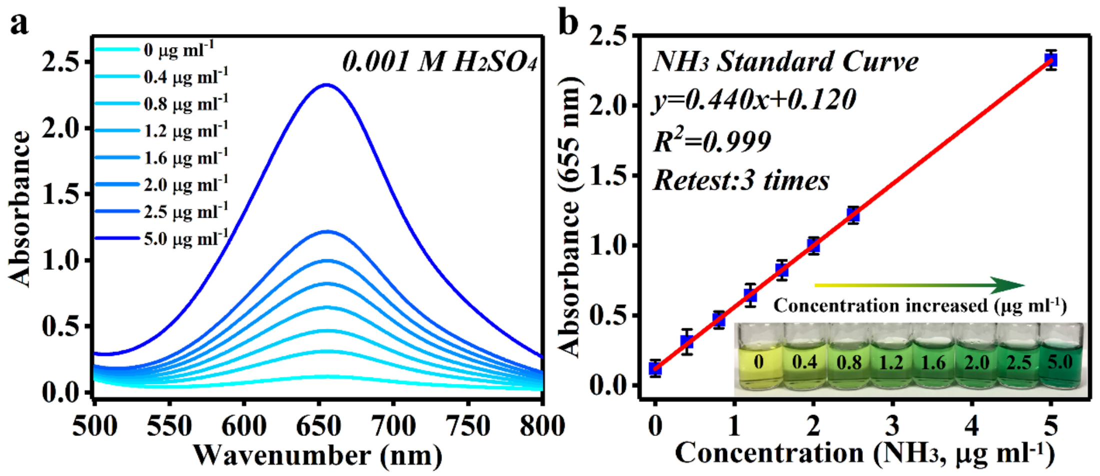

Supplementary Figure 7. Determination of the produced ammonia in 0.001 M H_2SO_4. **a** The UV-Vis absorption spectra and **b** corresponding calibration curves for the colorimetric NH_3 assay using the indophenol blue method in 0.001 M H_2SO_4.

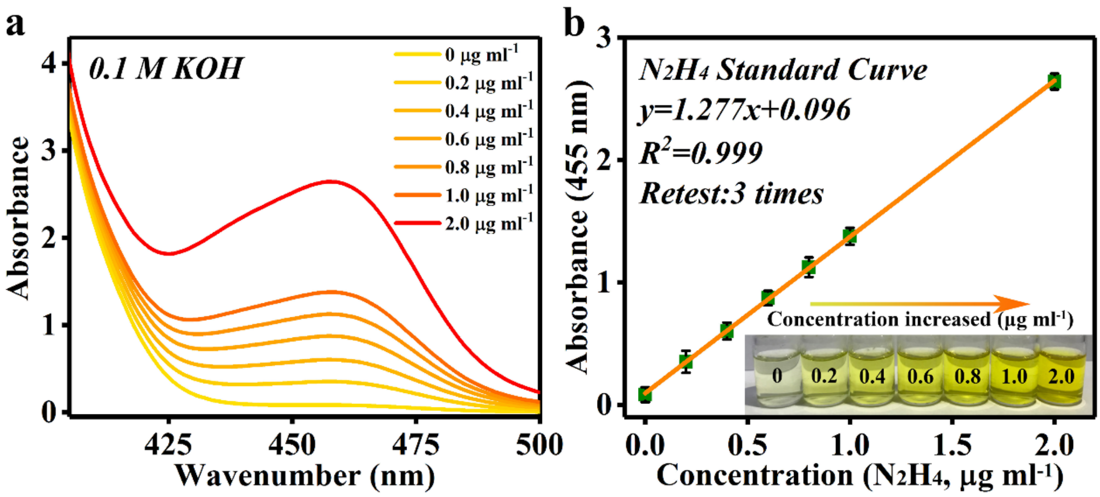

Supplementary Figure 8. Determination of the produced hydrazine in 0.1 M KOH. **a** The UV-Vis absorption spectra and **b** corresponding calibration curves for the colorimetric N_2H_4 assay using the indophenol blue method in 0.1 M KOH.

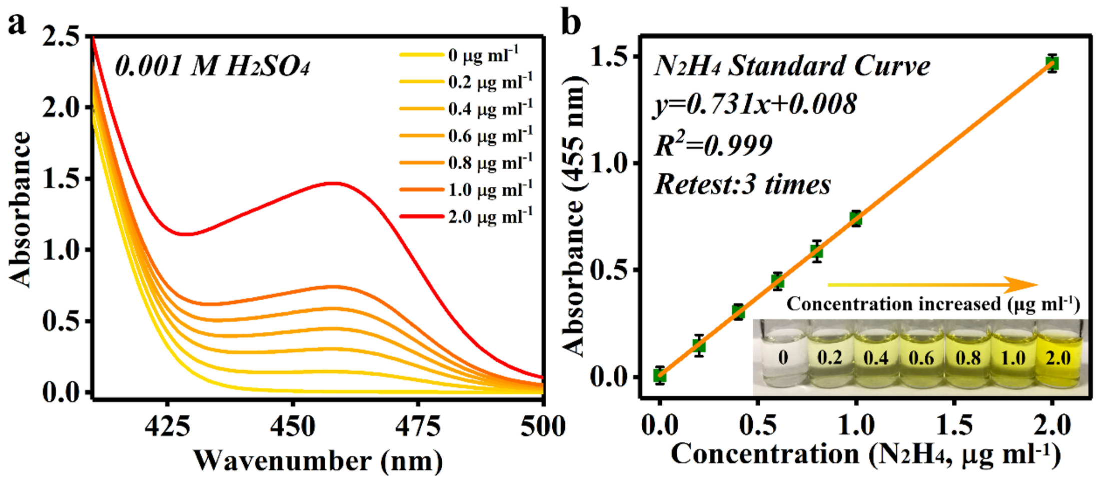

Supplementary Figure 9. Determination of the produced hydrazine in 0.001 M H_2SO_4. **a** The UV-Vis absorption spectra and **b** corresponding calibration curves for the colorimetric N_2H_4 assay using the indophenol blue method in 0.001 M H_2SO_4.

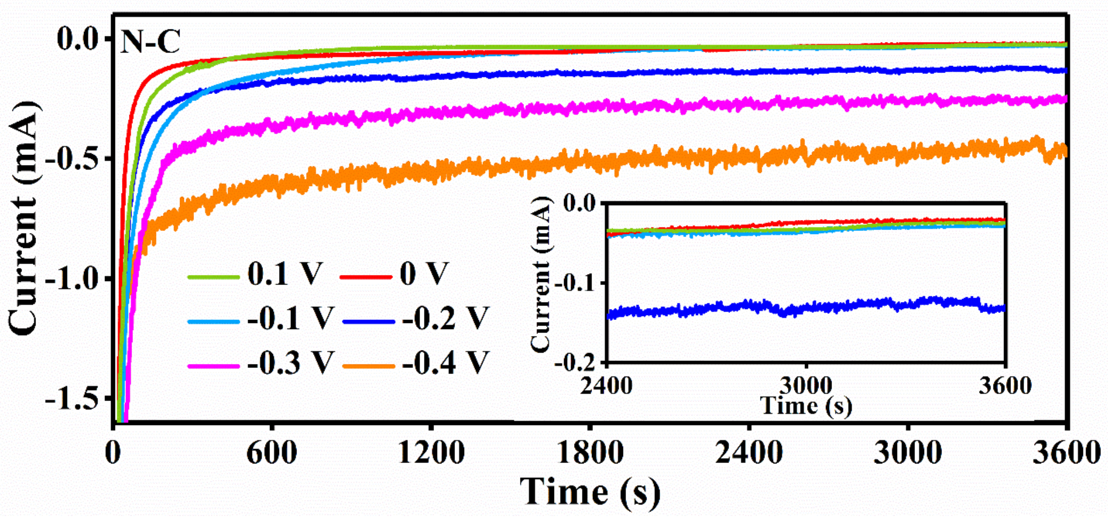

Supplementary Figure 10. Chronoamperometry results of N-C at the corresponding potentials, inset: enlarged view of the curves from -0.2 to 0.1 V vs. RHE.

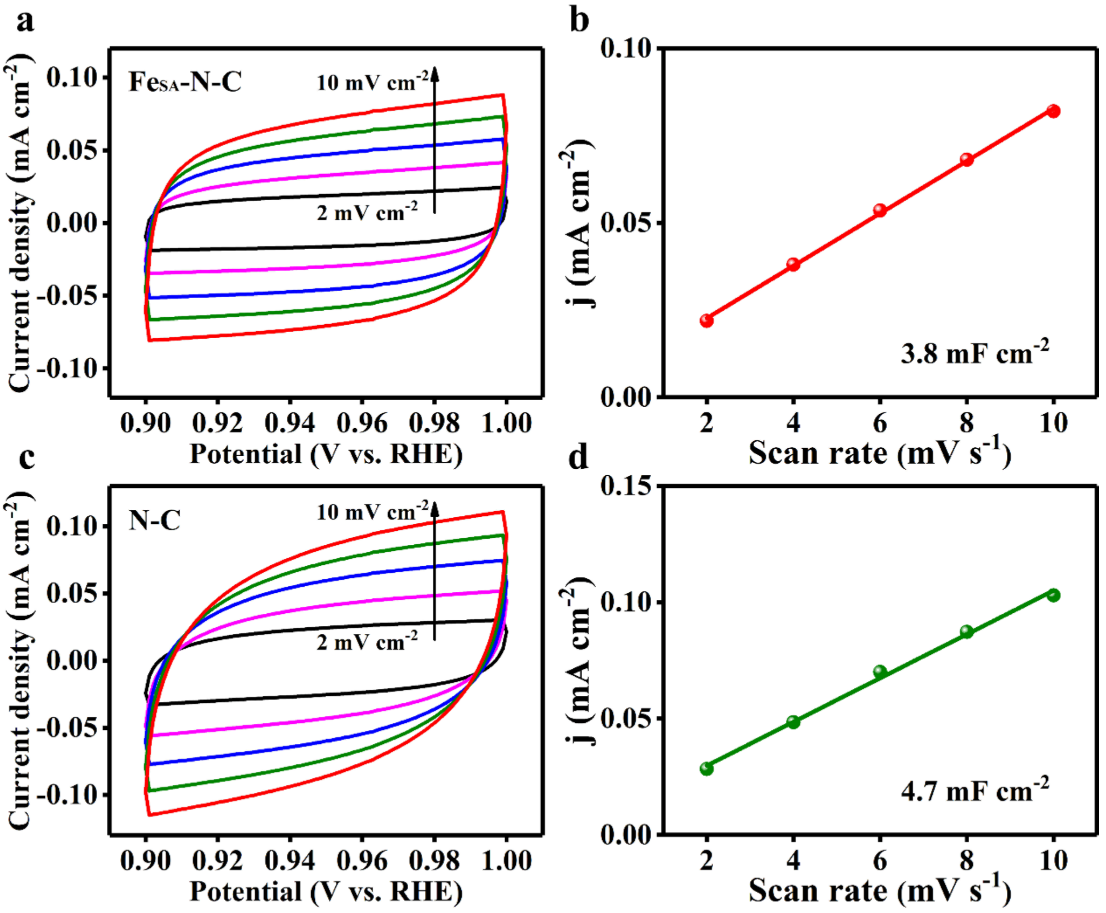

Supplementary Figure 11. The cyclic voltammograms (CVs) used to determine the electrochemical surface area (ECSA) for measuring the electrochemical double-layer capacitance (C_{dl}) of the materials. Cyclic voltammetry curves of **a** Fe$_{SA}$-N-C and **c** N-C measured at different scan rates from 2 to 10 mV s^{-1}. **b** and **d** Corresponding plots of the current density at 0.98 V vs. the scan rate.

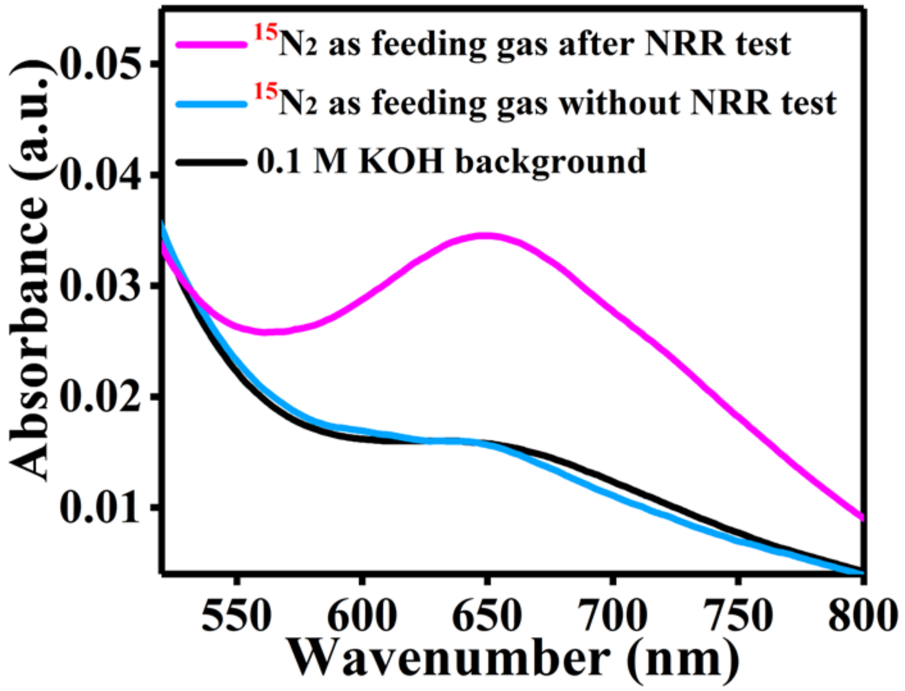

Supplementary Figure 12. UV-vis absorption spectra of the electrolytes after electrolysis at 0 V vs. RHE for 1 h using $^{15}N_2$ as feeding gas under different conditions.

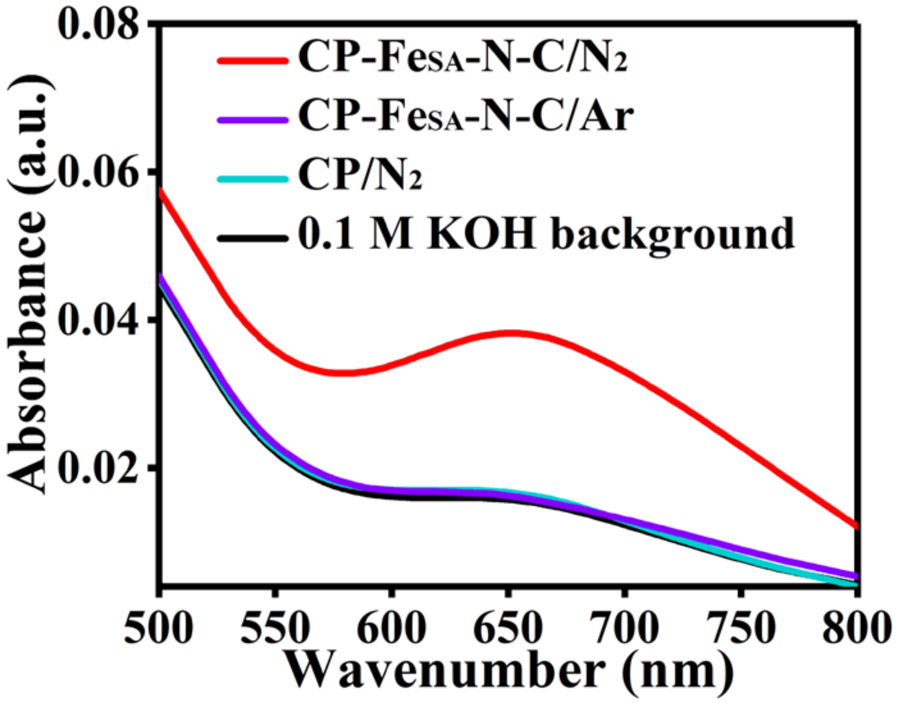

Supplementary Figure 13. UV-vis absorption spectra of the electrolytes after electrolysis at 0 V vs. RHE for 1 h under different conditions. No apparent NH_3 was detected for the control experiments with Ar-saturated electrolyte (CP-Fe_{SA}-N-C/Ar) or without Fe_{SA}-N-C catalyst (CP/N_2), indicating that NH_3 was produced by Fe_{SA}-N-C-catalyzed electroreduction of N_2.

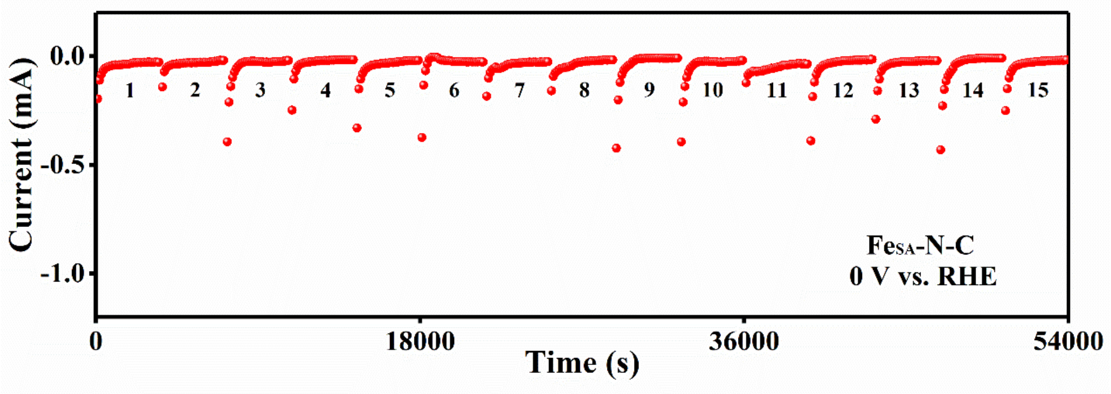

Supplementary Figure 14. Stability test of the Fe_{SA}-N-C catalyst in N_2-saturated 0.1 M KOH at 0 V vs. RHE under consecutive recycling electrolysis.

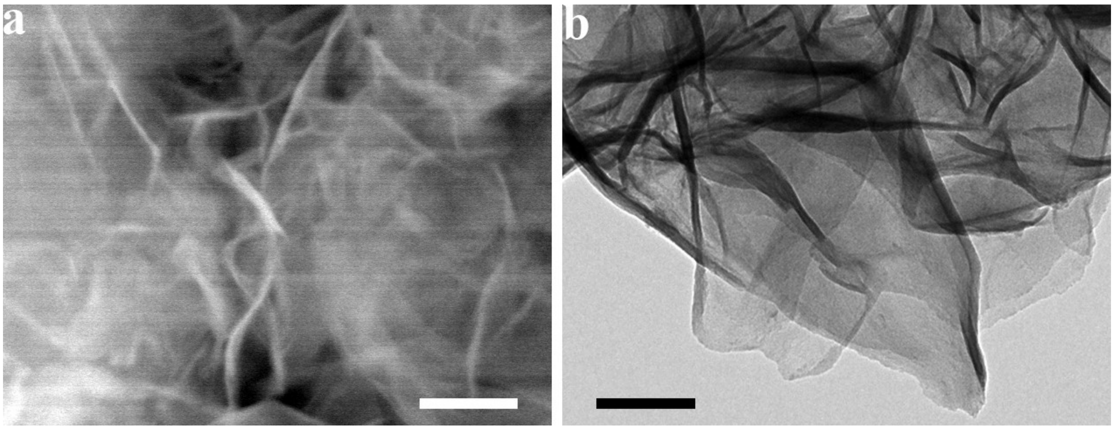

Supplementary Figure 15. Morphology characterization of the Fe_{SA}-N-C after NRR. **a** SEM image of the Fe_{SA}-N-C catalyst after NRR test. Scale bar, 200 nm. **b** TEM image of the Fe_{SA}-N-C catalyst after NRR test. Scale bar, 100 nm. The structure remains unchanged.

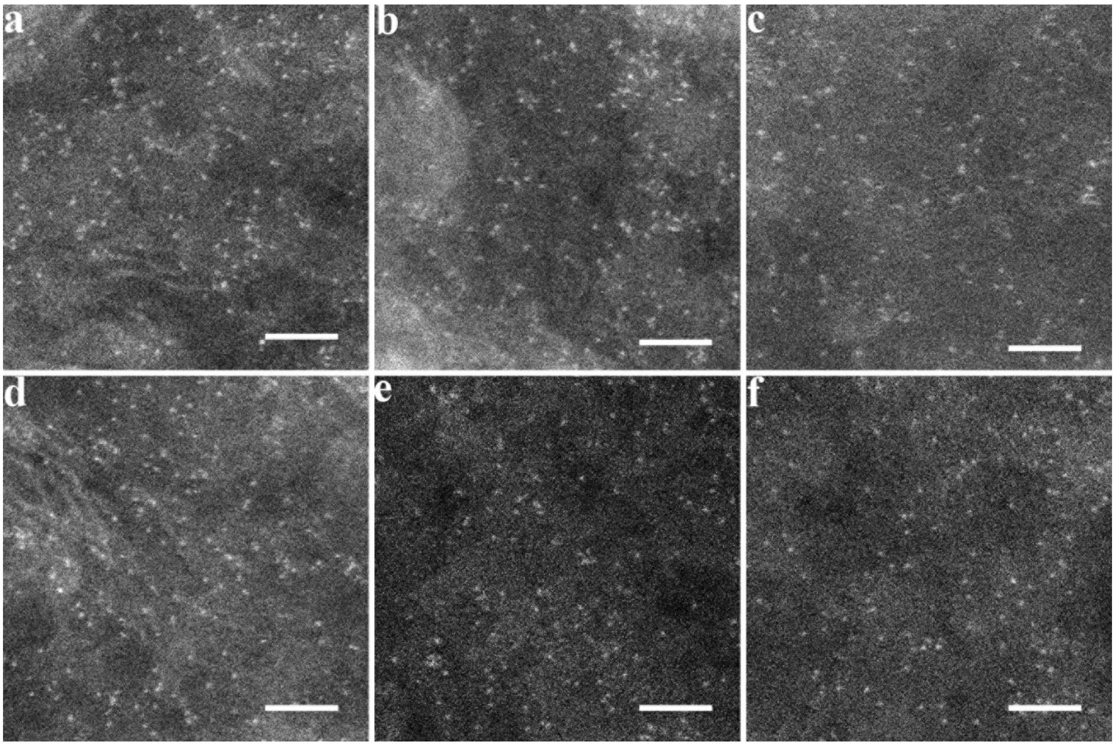

Supplementary Figure 16. Six representative HAADF-STEM images of the Fe_{SA}-N-C catalyst after NRR test, demonstrating the excellent stability of the atomically dispersed Fe atoms. Scale bar, 2 nm.

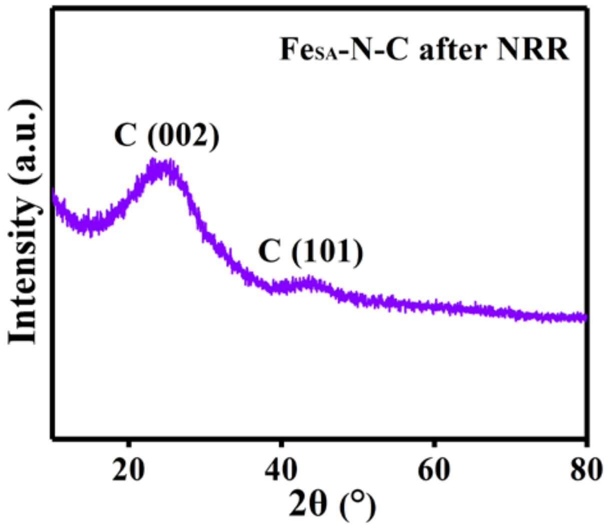

Supplementary Figure 17. XRD pattern for the Fe$_{SA}$-N-C catalyst after NRR test. Its phase property also shows no obvious change.

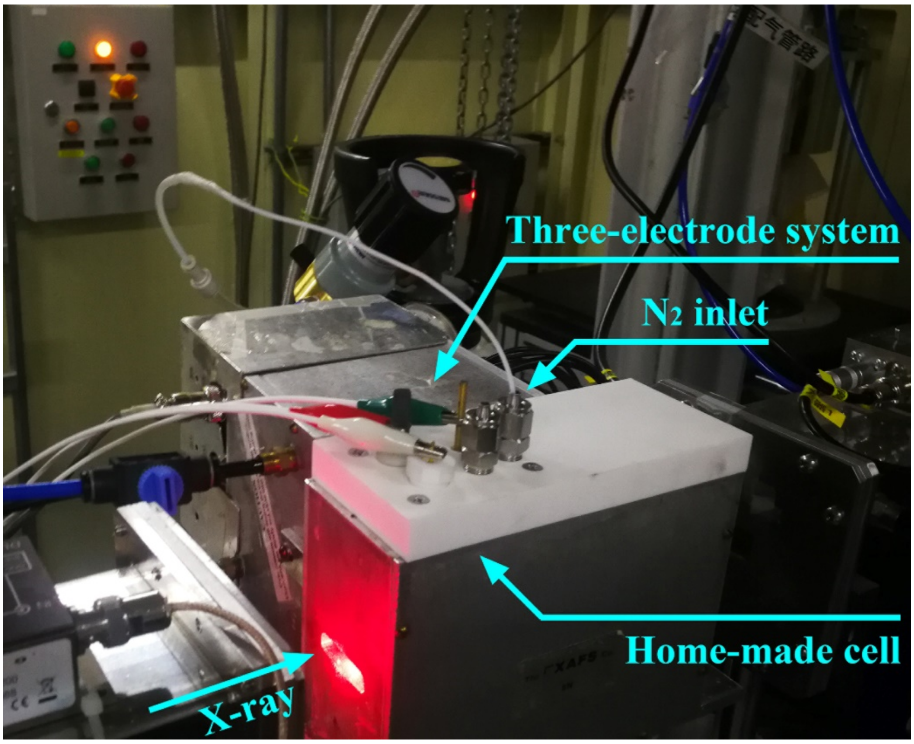

Supplementary Figure 18. Photograph of the equipment used for *in-situ* XAS characterization.

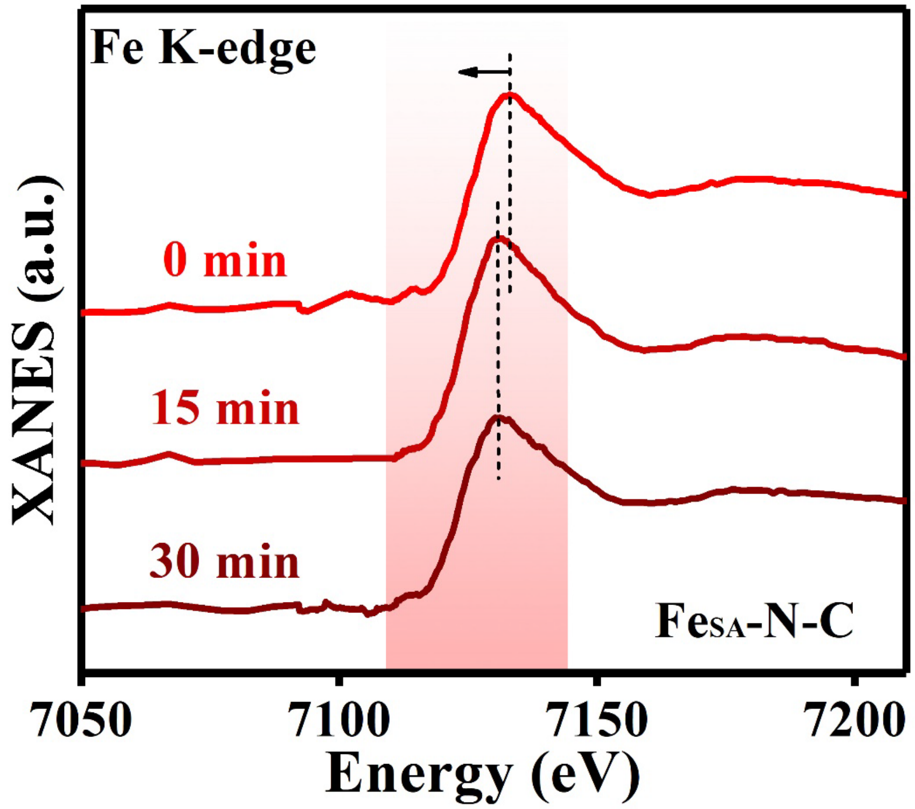

Supplementary Figure 19. *In-situ* XAS results of Fe$_{SA}$-N-C catalysts: Fe K-edge XANES spectra at 0 V vs. RHE as a function of time.

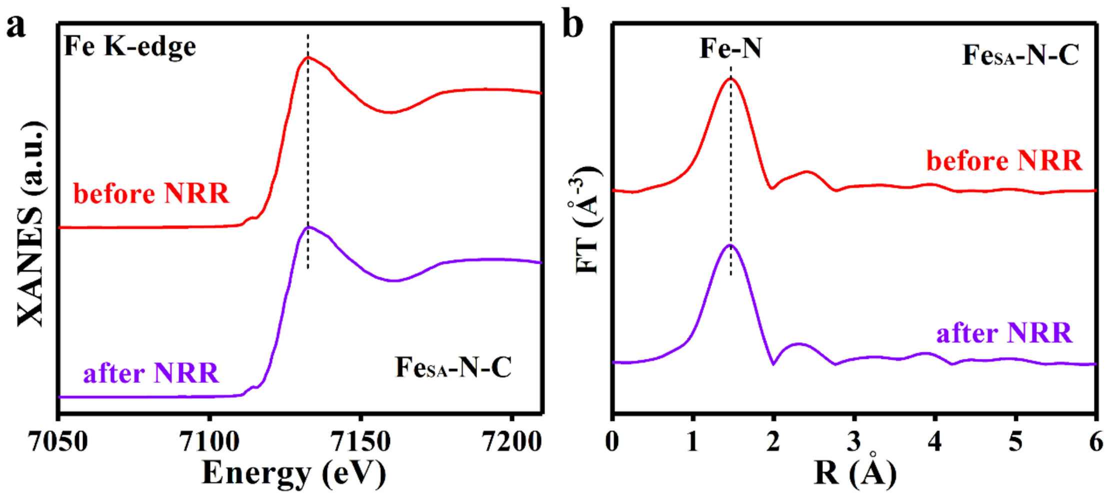

Supplementary Figure 20. *Ex-situ* XAS results of Fe_{SA}-N-C catalysts before and after NRR process: **a** XANES spectra and **b** Fourier transform spectra at the Fe K-edge.

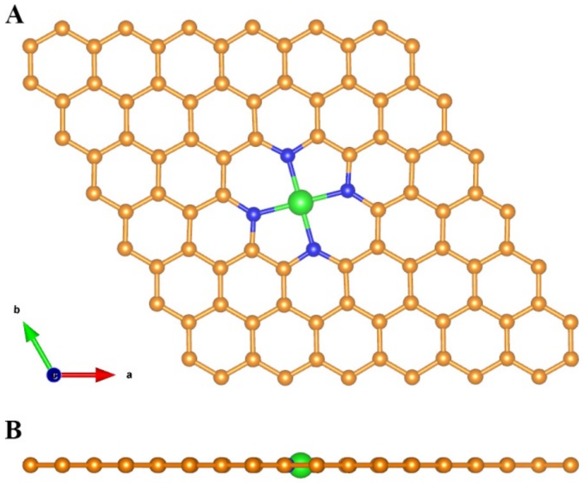

Supplementary Figure 21. Computational models of Fe_{SA}-N-C catalyst. **a** Top view. **b** Side view. The orange, blue, and green spheres represent C, N, and Fe atoms, respectively.

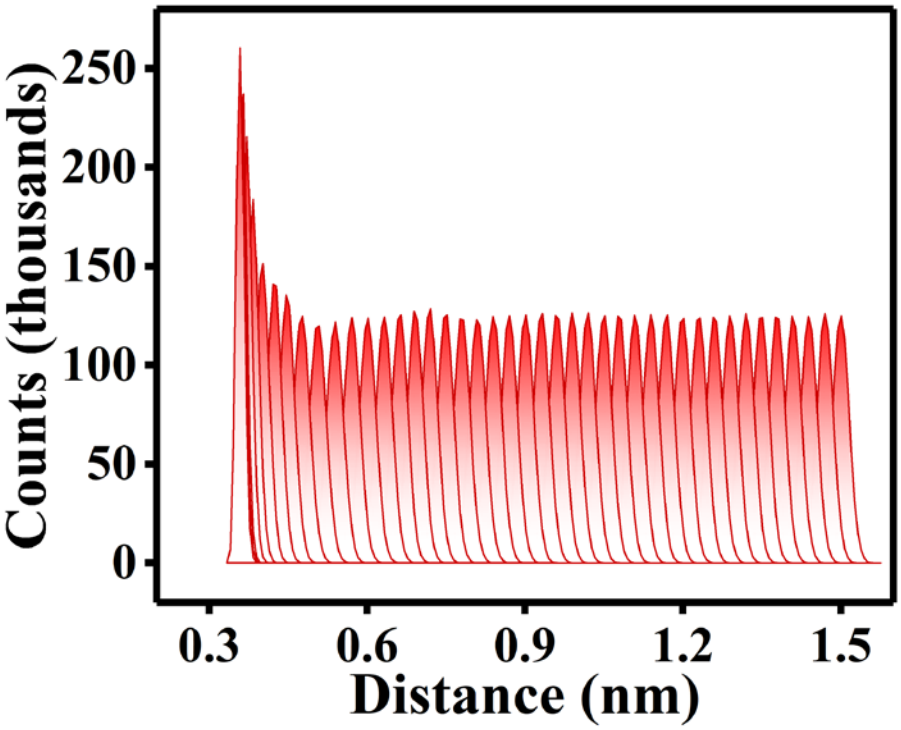

Supplementary Figure 22. Complete histograms of all window umbrella sampling statistics used for calculation of the N_2 adsorption on the Fe_{SA}-N-C catalyst.

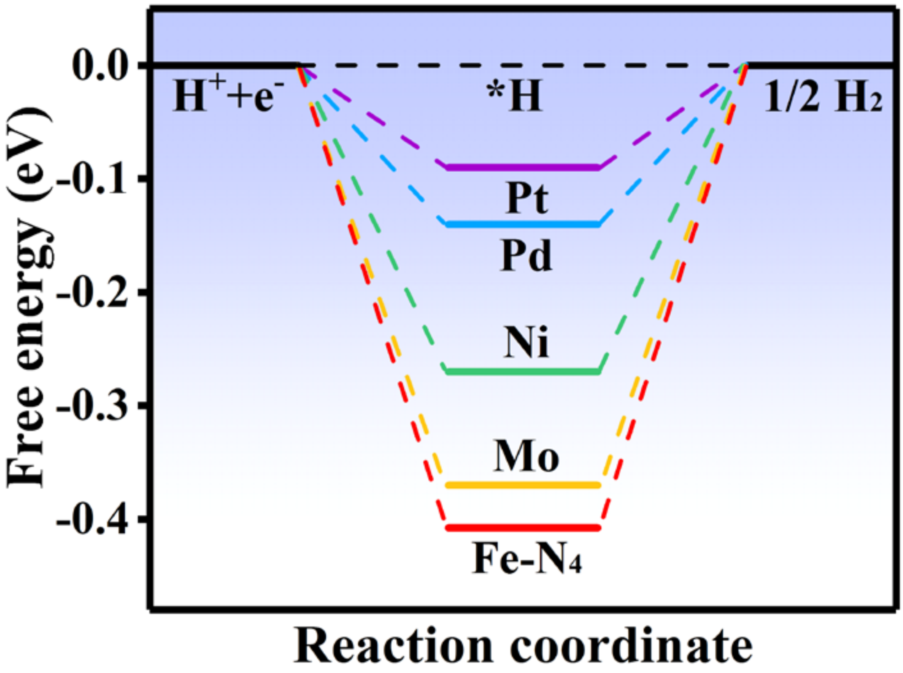

Supplementary Figure 23. Free energy diagrams for the HER on different models.

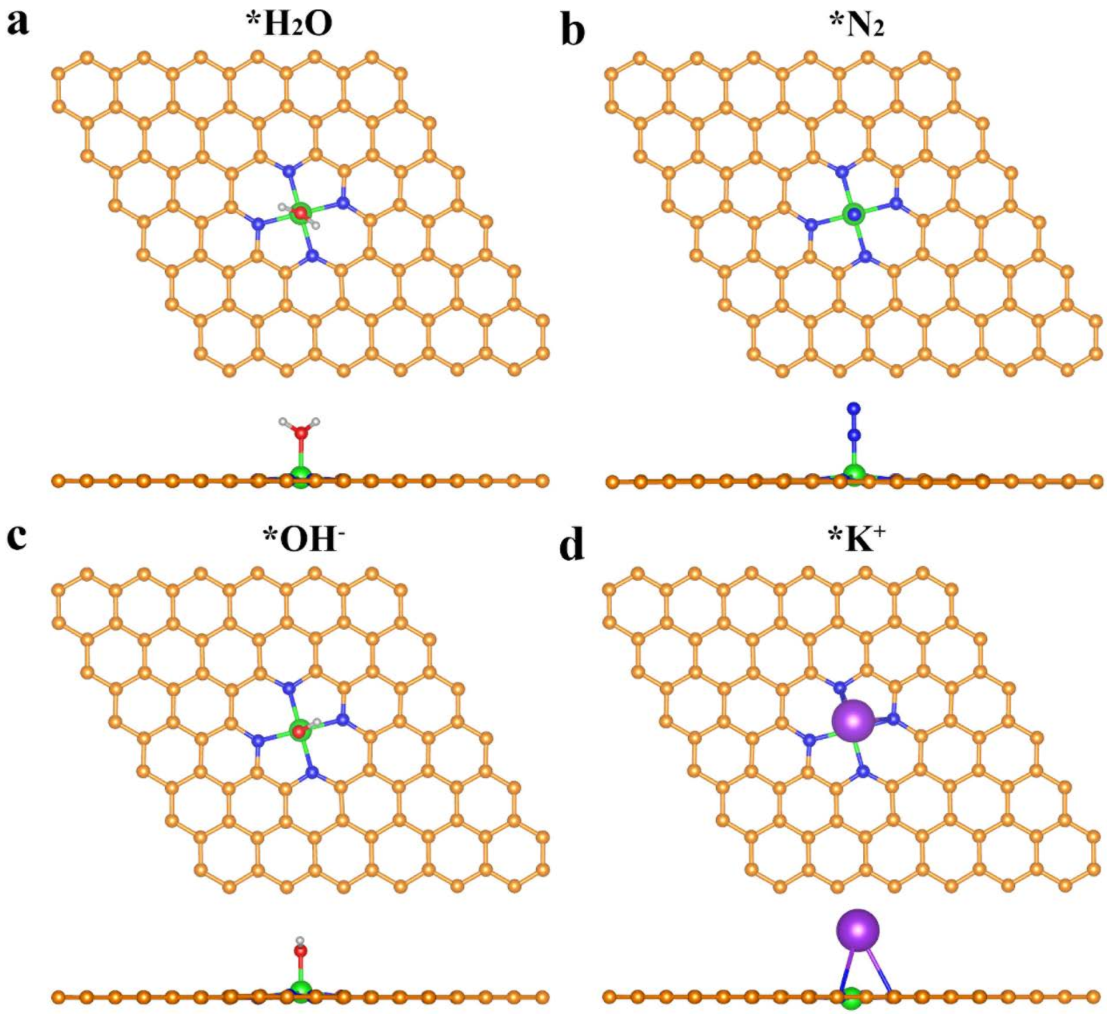

Supplementary Figure 24. Configurations of adsorption of **a** $*H_2O$, **b** $*N_2$, **c** $*OH^-$, and **d** $*K^+$ on Fe_{SA}-N-C catalyst. The orange, blue, red, grey, green, and purple spheres represent C, N, O, H, Fe, and K atoms, respectively.

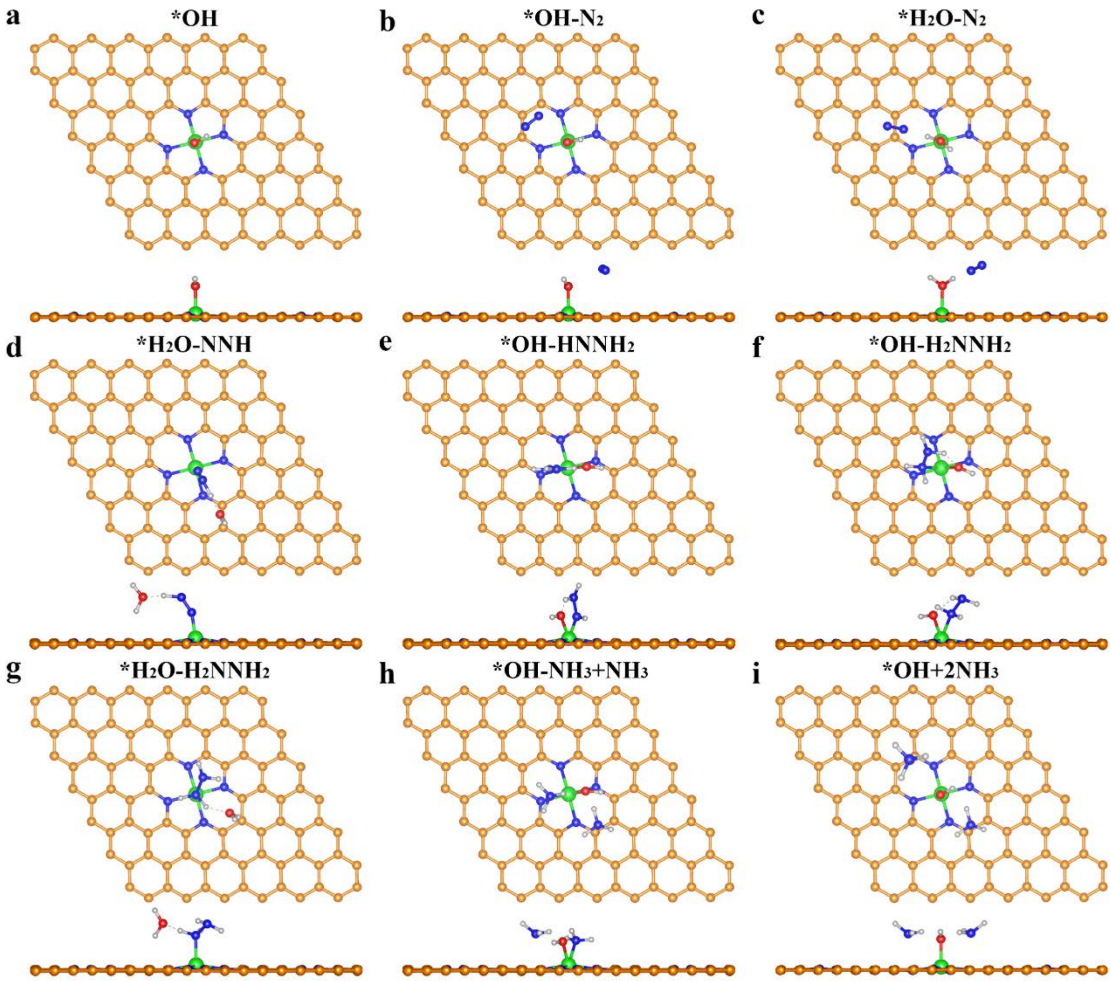

Supplementary Figure 25. Configurations of adsorbates on Fe_{SA}-N-C catalyst with an alternating pathway. The orange, blue, red, green, and grey spheres represent C, N, O, Fe, and H atoms, respectively.

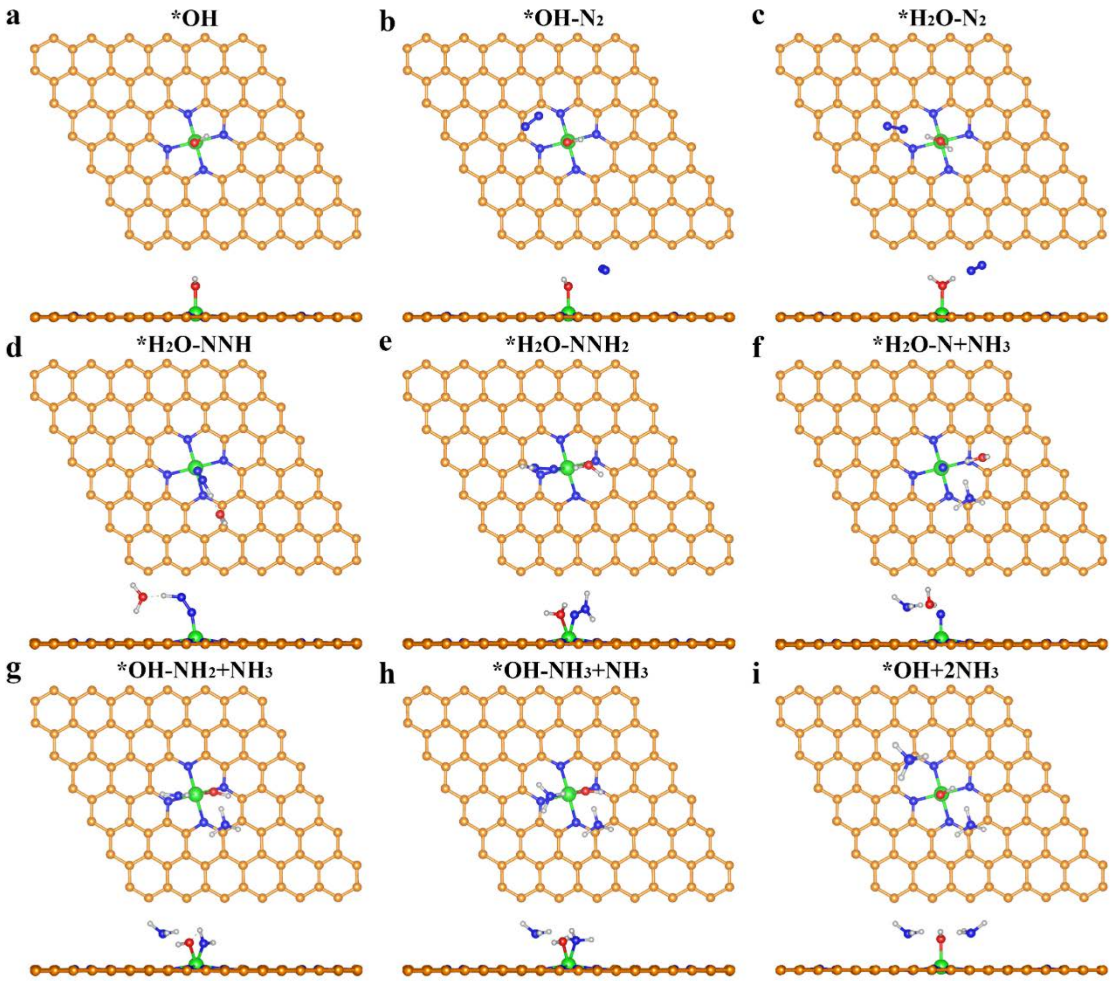

Supplementary Figure 26. Configurations of adsorbates on Fe_{SA}-N-C catalyst with a distal pathway. The orange, blue, red, green, and grey spheres represent C, N, O, Fe, and H atoms, respectively.

Supplementary Table 1. Comparison of the NRR performance of the Fe_{SA}-N-C catalyst with other catalysts reported to date under ambient conditions (room temperature and atmospheric pressure).

Catalyst	Electrolyte	Potential (V vs. RHE)	Faradaic efficiency (%)	Yield rate (μg mg^{-1} h^{-1})	Ref.
**Noble metal/Rare-earth metal electrocatalyst**					
Carbon black-supported Pd nanoparticles	0.1 M PBS	0.1	8.2	4.4	1
Tetrahexahedral Au nanorods	0.1 M KOH	−0.2	3.88	6.042	2
Au sub-nanoclusters embedded on TiO_2	0.1 M HCl	−0.2	8.11	21.4	3
CeO_x-induced amorphization of Au nanoparticles anchored on reduced graphite oxide	0.1 M HCl	−0.2	10.1	8.3	4
Ultrafine $Pd_{0.2}Cu_{0.8}$ amorphous nanoclusters on reduced graphene oxide	0.1 M KOH	−0.2	4.52	1.66	5
Amorphous $Bi_4V_2O_{11}$-crystalline CeO_2 hybrid	0.1 M HCl	−0.2	10.16	23.21	6
**Transition metal/Metal-free electrocatalyst**					
Poly(N-ethyl-benzene-1,2,4,5-tetracarboxylic diimide) covered carbon cloth	0.5 M Li_2SO_4	−0.5	2.85	1.23	7
N-doped porous carbon-750 °C	0.05 M H_2SO_4	−0.9	1.42	23.8	8
Polymeric carbon nitride with nitrogen vacancies	0.1 M HCl	−0.2	11.59	8.09	9
**Fe_{SA}-N-C**	**0.1 M KOH**	**0**	**56.55**	**7.48**	**This work**

For noble metal and rare-earth metal catalysts, their prohibitive cost and scarce reserve make them have to deliver excellent performance in every way, including Faradaic efficiency and yield rate. As for transition metal and metal-free catalysts, since they have abundant supply and low cost, their Faradaic efficiency is thus of high importance. In this context, Fe_{SA}-N-C is clearly the one best at doing so.

Supplementary Table 2. The NH_3 production amount (μg) in the catholyte (0.1 M KOH, 30 ml) and absorber (0.001 M H_2SO_4, 30 ml) of Fe_{SA}-N-C and N-C under different applied potentials.

**Potential (V vs. RHE)**	**Fe_{SA}-N-C**		**N-C**	
	Catholyte	Absorber	Catholyte	Absorber
0.1	2.519	0.273	0.955	0
0	5.505	1.977	1.097	0.205
-0.1	4.794	0.955	2.235	0.273
-0.2	4.510	0.886	3.230	0.341
-0.3	4.368	0.750	2.803	0.205
-0.4	3.941	0.136	2.377	0.136

Supplementary Table 3. Adsorption energies of $*H_2O$, $*N_2$, $*OH^-$, and $*K^+$ on the Fe_{SA}-N-C catalyst.

Species	Adsorption energy (eV)
$*H_2O$	1.2682
$*N_2$	-0.7445
$*OH^-$	-3.8404
$*K^+$	-2.2829

Supplementary References

1. Wang, J., Yu, L., Hu, L., Chen, G., Xin, H. & Feng, X. Ambient ammonia synthesis via palladium-catalyzed electrohydrogenation of dinitrogen at low overpotential. *Nat. Commun.* **9**, 1795–1801 (2018).

2. Bao, D., Zhang, Q., Meng, F. –L., Zhong, H. –X., Shi, M. –M., Zhang, Y., Yan, J. –M., Jiang, Q. & Zhang, X. –B. Electrochemical reduction of N_2 under ambient conditions for artificial N_2 fixation and renewable energy storage using N_2/NH_3 Cycle. *Adv. Mater.* **29**, 1604799 (2017).

3. Shi, M. –M., Bao, D., Wulan, B. –R., Li, Y. –H., Zhang, Y. –F., Yan, J. –M., & Jiang, Q. Au sub-nanoclusters on TiO_2 toward highly efficient and selective electrocatalyst for N_2 conversion to NH_3 at ambient conditions. *Adv. Mater.* **29**, 1606550 (2017).

4. Li, S. –J., Bao, D., Shi, M. –M., Wulan, B. –R., Yan, J. –M. & Jiang, Q. Amorphizing of Au nanoparticles by CeO_x–RGO hybrid support towards highly efficient electrocatalyst for N_2 reduction under ambient conditions. *Adv. Mater.* **29**, 1700001 (2017).

5. Shi, M. –M., Bao, D., Li, S. –J., Wulan, B. –R., Yan, J. –M. & Jiang, Q. Anchoring PdCu amorphous nanocluster on graphene for electrochemical reduction of N_2 to NH_3 under ambient conditions in aqueous solution. *Adv. Energy Mater.* **8**, 1800124 (2018).

6. Lv, C., Yan, C., Chen, G., Ding, Y., Sun, J., Zhou, Y. & Yu, G. An amorphous noble-metal-free electrocatalyst that enables nitrogen fixation under ambient conditions. *Angew. Chem. Int. Ed.* **130**, 6181–6184 (2017).

7. Chen, G. –F, Cao, X., Wu, S., Zeng, X., Ding, L. –X., Zhu, M. & Wang, H. Ammonia electrosynthesis with high selectivity under ambient conditions via a Li^+ incorporation strategy. *J. Am. Chem. Soc.* **139**, 9771–9774 (2017).

8. Liu, Y., Su, Y., Quan, X., Fan, X., Chen, S., Yu, H., Zhao, H., Zhang, Y. & Zhao, J. Facile ammonia synthesis from electrocatalytic N_2 reduction under ambient conditions on N-doped porous carbon. *ACS Catal.* **8**, 1186−1191 (2018).

9. Lv, C., Qian, Y., Yan, C., Ding, Y., Liu, Y., Chen, G. & Yu, G. Defect engineering metal-free polymeric carbon nitride electrocatalyst for effective nitrogen fixation under ambient conditions. *Angew. Chem. Int. Ed.* **57**, 10246–10250 (2018).
